# *AtGCS* promoter-driven clustered regularly interspaced short palindromic repeats/Cas9 highly efficiently generates homozygous/biallelic mutations in the transformed roots by *Agrobacterium rhizogenes*–mediated transformation

**DOI:** 10.3389/fpls.2022.952428

**Published:** 2022-10-18

**Authors:** Shuang Liu, Xiuyuan Wang, Qianqian Li, Wentao Peng, Zunmian Zhang, Pengfei Chu, Shangjing Guo, Yinglun Fan, Shanhua Lyu

**Affiliations:** College of Agriculture, Liaocheng University, Liaocheng, China

**Keywords:** CRISPR/Cas9, *agrobacterium rhizogenes*–mediated transformation (ARM), genome editing, homozygous/biallelic mutation, hairy root, gamma-*glutamylcysteine synthetase* gene

## Abstract

*Agrobacterium rhizogenes*–mediated (ARM) transformation is an efficient and powerful tool to generate transgenic roots to study root-related biology. For loss-of-function studies, transgenic-root-induced indel mutations by CRISPR/Cas9 only with homozygous/biallelic mutagenesis can exhibit mutant phenotype(s) (excluding recessive traits). However, a low frequency of homozygous mutants was produced by a constitutive promoter to drive *Cas9* expression. Here, we identified a highly efficient *Arabidopsis thaliana* gamma-*glutamylcysteine synthetase* promoter, termed *AtGCSpro*, with strong activity in the region where the root meristem will initiate and in the whole roots in broad eudicots species. *AtGCSpro* achieved higher homozygous/biallelic mutation efficiency than the most widely used *CaMV 35S* promoter in driving *Cas9* expression in soybean, *Lotus japonicus*, and tomato roots. Using the p*AtGCSpro*-Cas9 system, the average homozygous/biallelic mutation frequency is 1.7-fold and 8.3-fold higher than the p*2 × 35Spro*-Cas9 system for single and two target site(s) in the genome, respectively. Our results demonstrate the advantage of the p*AtGCSpro*-Cas9 system used in ARM transformation, especially its great potential in diploids with multiple-copy genes targeted mutations and polyploid plants with multiplex genome editing. *AtGCSpro* is conservatively active in various eudicots species, suggesting that *AtGCSpro* might be applied in a wide range of dicots species.

## Introduction

*Agrobacterium rhizogenes*–mediated (ARM) transformation has revolutionized biological research through its ability to rapidly, simply, and conveniently generate transgenic roots of plant species, including in species recalcitrant to genetic transformation mediated by *A. tumefaciens* (Fan et al., 2020a; Fan et al., 2020b). Transgenic hairy roots co-transformed with the T-DNA from both the Ri plasmid of *A*. *rhizogenes* (carrying *root locus* [*rol*] genes, inducing the production of hairy roots) and the binary vector (Chilton et al., 1982; Irigoyen et al., 2020) can be generated. ARM transformation has already been established in a wide variety of plant taxa of more than 100 species and has widely been used for the modification of root traits, either because no protocols for stable *A. tumefaciens*–mediated transformation to generate transgenic plant (whole plant is genetically modified) or because root-related biological traits were analyzed. The composite plant generated by ARM transformation is composed of transgenic roots and wild shoot, which has already broadly applied for interactions between roots and microbes (e.g., rhizobia, arbuscular mycorrhizal fungi, pathogens, and nematode), signal transduction between root and shoot, and interactions between plant roots and environment (biotic/abiotic stresses). In addition, transgenic hairy roots can be rapidly induced and produced higher biomass by ARM transformation for the fast production of secondary metabolites and phytoremediation (e.g., Plasencia et al., 2016; Fan et al., 2017; Wang et al., 2017; Yang et al., 2017; Jenei et al., 2020; Fan et al., 2020a; Zhang et al., 2021).

To knock out gene(s) for loss-of-function studies in roots by ARM transformation, clustered regularly interspaced short palindromic repeats (CRISPR)–associated Cas (CRISPR/Cas) systems provide a convenient and powerful tool. The CRISPR/Cas9 system is the most frequently and widely employed targeted genome editing tool due to its simplicity, high specificity, efficiency, and multiplexing capacity (Hua et al., 2019). The CRISPR/Cas9 system is composed of the single-guide RNA (sgRNA) for target DNA recognition and the Cas9 nuclease for DNA cleavage. Previous studies had shown that the editing efficiency and mutation types (homozygous, heterozygous, or bi-allelic) mediated by the CRISPR/Cas9 system varied considerably in different plant tissues and species when different promoters were used to drive the expression of *Cas9 via A*. *tumefaciens*–mediated stable genetic transformation (Wang et al., 2015; Yan et al., 2015; Eid et al., 2016; Gao et al., 2016; Mao et al., 2016; Tsutsui and Higashiyama, 2017; Feng et al., 2018). However, genome editing efficiencies mediated by CRISPR/Cas9 using different promoters to drive the expression of *Cas9* by ARM transient transformation have not been evaluated. In most cases, cauliflower mosaic virus (CaMV) *35S* is used to drive the expression of *Cas9* (Yan et al., 2015; Tang et al., 2016; Ma et al., 2016; Fan et al., 2017; Feng et al., 2018; Zhang et al., 2020) in ARM transformation but with a low genome editing efficiency. In the transformed hairy roots mediated by ARM transformation, except for recessive traits, mutant phenotype(s) can be observed only when all alleles are edited and homozygous/biallelic mutations (H/BM) generated. This is a challenge and bottleneck to achieving multiple targeted loci simultaneous homozygous/biallelic mutagenesis in the transient expression of the CRISPR/Cas9 system in diploid and polyploid plants with multiple gene copies. Large numbers of transformants need to be selected and further identified whether homozygous/biallelic mutagenesis was induced at all the target sites, which is labor intensive, time consuming, tedious, and costly.

Here, we describe a highly efficient *Arabidopsis thaliana* gamma-*glutamylcysteine synthetase* promoter named *AtGCSpro* (7-GCS; EC 6.3.2.2; May and Leaver, 1994). p*AtGCSpro*::*GUSPlus* transformed whole roots showed a GUS signal and a high level of GUS activity in the initiation region of root meristem undergoing active cell division in broad eudicots diploid soybean (*Glycine max*), tomato (*Solanum lycopersicum*), cucumber (*Cucumis sativus* L.), *Lotus japonicus*, as well as in polyploid tobacco (*Nicotiana tabacum* L.), cotton (*Gossypium* spp), and sweet potato (*Ipomoea batatas*). Our results indicate the advantage of using *AtGCSpro* for CRISPR/Cas9 genome editing in inducing H/BM rate applied in ARM transformation of *L. japonicus* (a model leguminous plant species), soybean, and tomato. This approach has great potential in research addressing multiplex gene copies or gene families with functional redundancy. The conserved and high activity of *AtGCSpro* in roots covering a wide range of dicots species suggests that *AtGCSpro* might have great potential to be applied broadly to achieve high H/BM rates at target sites by CRISPR/Cas9 *via* ARM transformation.

## Materials and methods

### Plant materials and growth conditions

Soybean (*Glycine max*) Williams 82, *L. japonicus* (*Gifu-*129), cucumber (*Cucumis sativus* L.) “Chinese long” inbred line 9930, tomato (*Solanum lycopersicum*) local variety Maofeng802, sweet potato (*Ipomoea batatas*) local variety Jishu25, cotton (*Gossypium* spp.) local variety Lumianyan28, tobacco (*Nicotiana benthamiana*), and *Arabidopsis thaliana* Columbia (Col-0) were used in this study. The plants were grown in a greenhouse under a photoperiod of 16h light (80 µM photons m^-2^ s^-1^)/8h dark at 24 ± 2C.

### Cloning of *AtGCSpro*, construction of various lengths *AtGCSpro* to drive the expression of *GUSPlus* plasmid vectors, histological GUS staining, and qRT-PCR analysis

To isolate *AtGCSpro*, a 2411-bp upstream promoter region of the translation start site of gamma-*glutamylcysteine synthetase* gene (GenBank accession no. AF068299.1) was amplified by PCR using a GaBa1 primer containing a *Bam*HI site combined with a GaBNR primer containing a *Bsa*I site (produced 5′-CATG sticky end) from *A. thaliana* Columbia (Col-0) genomic DNA as a template. All primers sequences used in this paper are listed in **Supplementary Table S1**.

**Figure 1:**
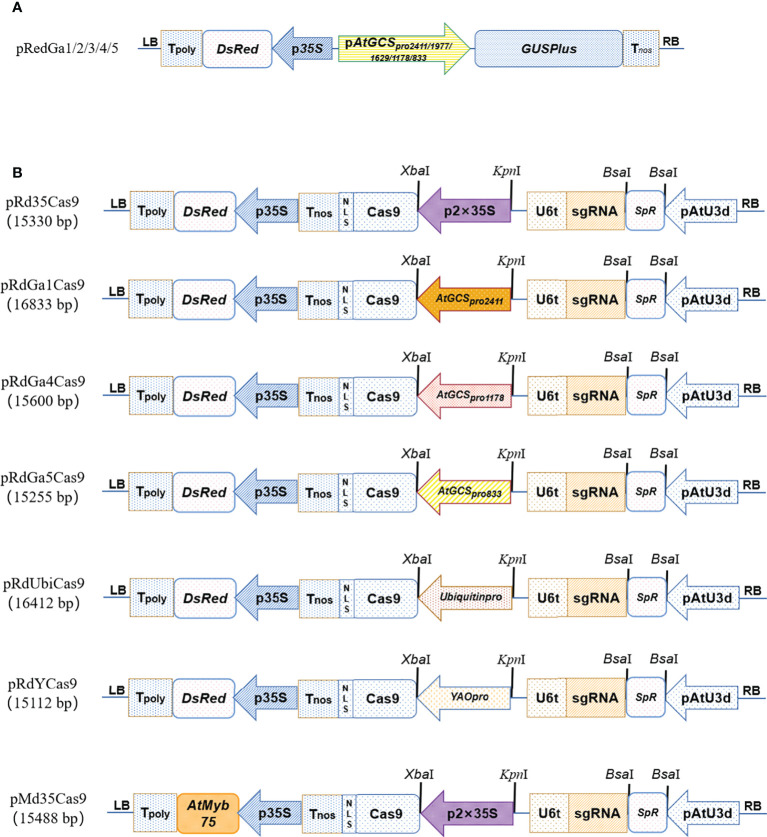
The schematic diagrams of vectors. The schematic diagrams of the pRedGa1 (*AtGCSpro*_2411_:: *GUSPlus*), pRedGa2 (*AtGCSpro*_1977_:: *GUSPlus*), pRedGa3 (*AtGCSpro*_1629_:: *GUSPlus*), pRedGa4 (*AtGCSpro*_1178_:: *GUSPlus*), and pRedGa5 (*AtGCSpro*_833_:: *GUSPlus*) **(A)**. CRISPR/Cas9-mediated gene knockout vector backbones pRd35Cas9, pRdGa1Cas9, pRdGa4Cas9, pRdGa5Cas9, pRdUbiCas9, pRdYCas9, and pMd35Cas9 **(B)**.

To generate *GUSPlus* expressing vectors with various *AtGCSpro* promoter lengths, a recombinant binary vector pRed1305 (Fan et al., 2020b) harboring a *GUS* gene driven by CaMV35S with an intron from the *catalase* gene was used as the backbone. The CaMV35S promoter in pRed1305 was replaced by various lengths of *AtGCSpro*, respectively. Shortened lengths of *AtGCSpro* were PCR-amplified from the 2411-bp *AtGCSpro* with a reverse primer (GaBNR) and a forward primer (GaBa2, GaBa3, GaBa4, or GaBa5). The PCR amplification products, including a *Bam*HI restriction site at the 5′ end and a *Bsa*I restriction site at the 3′ end, were digested and directly ligated into pRed1305 previously digested with *Bam*HI and *Nco*I, thus producing the pRedGa1 (*AtGCSpro*_2411_:: *GUSPlus*), pRedGa2 (*AtGCSpro*_1977_:: *GUSPlus*), pRedGa3 (*AtGCSpro*_1629_:: *GUSPlus*), pRedGa4 (*AtGCSpro*_1178_:: *GUSPlus*), and pRedGa5 (*AtGCSpro*_833_:: *GUSPlus*) vectors. Schematic diagrams of the constructs are shown in **Figure 1A**. All constructs mentioned in the paper were confirmed by Sanger sequencing. The DNA ladder DL2000 in this paper was bought from Sangon Biotech (China, Shanghai).

Histological GUS staining was performed as previously described (Fan et al., 2020b). Relative expression levels of *GUS* were performed by a quantitative Real-time PCR (qRT-PCR) assay according to Lü et al. (2010) with the following minor modifications. The amplification of the soybean *GmActin* gene was used for normalization, and the primer pairs GmActinF and GmActinR were used according to Fan et al. (2020a). The gene-specific primer pairs GUSPF and GUSPR for *GUSPlus* gene were used. qRT-PCR experiments were performed with three replicates. In a biological replicate, for each independent transformed event (transformed pRedGa1, pRedGa2, pRedGa3, pRedGa4, or pRedGa5 construct), total 10 independent transgenic positive roots (with ~4 cm root lengths) for each independent construct were individually sampled, ground in liquid nitrogen, and used for total RNA extraction, respectively.

### Construction of CRISPR/Cas9-mediated gene knockout vector backbones

We first generated a series of CRISPR/Cas9-mediated gene knockout vector backbones: pRd35Cas9 (p*2×35Spro*-Cas9), pRdGa1Cas9 (p*AtGCSpro*_2411_-Cas9), pRdGa4Cas9 (p*AtGCSpro*_1178_-Cas9), pRdGa5Cas9 (p*AtGCSpro*_833_-Cas9), pRdUbiCas9 (p*Ubiquitin_pro_*-Cas9), pRdYCas9 (p*YAO_pro_*-Cas9), and pMd35Cas9 (**Figure 1B**).

We first recombined an intermediate vector pRSE401 (**Supplementary Figure S1**) based on the backbone of pHSE401 (Xing et al., 2014), a *DsRed* reporter gene driven by CaMV35S promoter that replaced the *Hpt II* (*Hygromycin Phosphotransferase II*), which can be easily screened for transgenic positive hairy roots (**Supplementary Figure S2**). To introduce the *DsRed* driven by the CaMV35S promoter into pHSE401, the p35S-*DsRed*-CaMV poly(A)-LB cassettes (primary regions from 8,392 to 11,034 in pCAMBIA1305) were produced from the vector pRed1305 (Fan et al., 2020b) digested by *Eco*RI and *Sac*II and then inserted into pHSE401.

In addition, to shorten the vector sizes, the AtU6-26 promoter (424 bp) was replaced by the AtU3d promoter (121 bp) to drive the gRNA expression (Ma et al., 2015). The complete gRNA expression cassettes of AtU3d-gRNA-Sc-U6-26t were generated by recombinant PCR. To substitute AtU6-26 for AtU3d in pRSE401, the AtU3d promoter (primary regions 144–264 bp in pYLsgRNA-AtU3d) was amplified by PCR with primers Sap401 and Rd4012 using pYLsgRNA-AtU3d plasmid (Ma et al., 2015) as the template. *Bsa*I-gRNA-Sc-U6-26t cassettes (primary regions 1327–2190 bp in pHSE401, Addgene No. 62201) were amplified by PCR with primers Rd4013 and Sap402 using pHSE401 plasmid (Ma et al., 2015) as the template. The two PCR fragments were recombined to generate the sgRNA expression cassettes of AtU3d-*Bsa*I-SpR-*Bsa*I-gRNA-Sc-U6-26t by recombinant PCR using primers Sap401 and Sap402, followed by digestion using *Sap*I, which was cloned into pRSE401 (**Supplementary Figure S1**) and pPG35Cas9 (Fan et al., 2020c), respectively, previously digested by *Hin*dIII followed by added with an “A” at the 3′ end of cohesive ends using KOD DNA polymerase with dATP. Therefore, the recombinant CRISPR-Cas9 vectors pRd35Cas9 and pMd35Cas9 were generated (**Figure 1B**). Based on the backbone of pRd35Cas9, an *AtGCSpro*_2411_ promoter replaced the 2 × 35S and produced the pRdGa1Cas9 (**Figure 1B**).

pRdGa1Cas9 generation was as follows. Full-length *AtGCSpro*_2411_ was amplified by PCR with primer GaK1 with a *Bsa*I restriction enzyme digestion site (produce 5′-GTAC sticky end) at the 5′ end and primer GaX2 with an *Xba*I at the 3′ end and then digested using restriction enzymes for cloning into pRd35Cas9 previously digested by Acc651 and *Xba*I and, therefore, produced vector pRdGa1Cas9 (**Figure 1B**).

The *Ubiquitin* promoter, *YAO* promoter, *AtGCSpro*_1178_, and *AtGCSpro*_833_ were amplified by PCR with the primers Ubikp/Ubixp (for *Ubiquitin_pro_*), YAOF18/PYao2 (for *YAO_pro_*), GaBa4/GaX2 (for *AtGCSpro*_1178_), and GaK5/GaX2 (for *AtGCSpro*_833_) using pYLCRISPR/Cas9Pubi-B (Ma et al., 2015), pYGUS1305 (Fan et al., 2020b), *AtGCSpro*_2411_, and *AtGCSpro*_2411_ as the templates, respectively, and digested by *Kpn*I/*Bsa*I, *Kpn*I/*Bsa*I, *Kpn*I/*Xba*I, and *Kpn*I/*Xba*I, respectively, for cloning into the *Kpn*I/*Xba*I restriction sites of the pRd35Cas9, and therefore generated pRdUbiCas9, pRdYCas9, pRdGa4Cas9, and pRdGa5Cas9 (**Figure 1B**).

### Construction of CRISPR/Cas9 genome editing vectors using different promoters to drive Cas9 expression

To construct genome editing vectors to knockout soybean *Rj7*, pRdGa1Cas9, pRdUbiCas9, pRdYCas9, pRdGa4Cas9, pRdGa5Cas9, and pRd35Cas9 were used as a backbone, respectively. Oligos Ktrj71 and Ktrj72 specifically targeted the soybean *Rj7* for construction of p*2×35Spro*-Cas9-*Rj7*, p*Ubiquitin_pro_*-Cas9-*Rj7*, p*YAOpro*-Cas9-*Rj7*, p*AtGCSpro*_2411_-Cas9-*Rj7*, p*AtGCSpro*_1178_-Cas9-*Rj7*, and *AtGCSpro*_833_-Cas9-*Rj7* vector, respectively.

To construct genome editing vectors to knockout *L. japonicus LjNLP4*, oligos KtLjNL1 and KtLjNL2 were designed and located in the exon of the open reading frame of *LjNLP4* (position: 28146509-28146531). The CRISPR/Cas9 vectors pMd35Cas9, pRdGa1Cas9, pRdUbiCas9, pRdYCas9, and pRdGa4Cas9 and pPG35Cas9 (**Figure 1B**) were used and generated the p*2×35Spro*-Cas9-*LjNLP4*, p*AtGCSpro*_2411_-Cas9-*LjNLP4*, p*Ubiquitin_pro_*-Cas9-*LjNLP4*, p*YAO_pro_*-Cas9-*LjNLP4*, and p*AtGCSpro*_1178_-Cas9-*LjNLP4* vectors, respectively.

To construct simultaneously targeting two genome sites, oligos ktGmR11 and ktGmR12 were designed to specifically target soybean *GmNNL1* (Zhang et al., 2021) and *Rfg1* (Fan et al., 2017) using pRd35Cas9 and pRdGa4Cas9 (**Figure 1B**) as backbones for generating p*2×35Spro*-Cas9-*GmNNL1Rfg1* and p*AtGCSpro*_1178_-Cas9-*GmNNL1Rfg1* vectors, respectively. Oligos ktLjSNF and ktLjSNR were designed to specifically target *L. japonicus LjNLP4* and *LjSYMRK* (Wang et al., 2016) using pMd35Cas9 and pRdGa4Cas9 (**Figure 1B**) as backbones for construction of the p*2×35Spro*-Cas9-*LjNLP4LjSYMRK* and *AtGCSpro*_1178_-Cas9-*LjNLP4LjSYMRK*, respectively. Oligos ktSlTRY1 and ktSlTRY2 were designed to specifically target two different sites within the first and second exon of tomato *SlTRY* (Tominaga-Wada et al., 2013), respectively, using pRd35Cas9 and pRdGa4Cas9 (**Figure 1B**) as backbones for generating p*2×35Spro*-Cas9-*SlTRY* and p*AtGCSpro*_1178_-Cas9-*SlTRY*, respectively. The Optimized CRISPR Plant Design Tool (http://cbi.hzau.edu.cn/cgi-bin/CRISPR) was used to design the oligos for constructing of CRISPR/Cas9 vector(s) (Lei et al., 2014). CRISPR/Cas9**-**mediated gene mutation vectors were constructed according to the procedure described previously by Xing et al. (2014). Specifically, for cloning a single gRNA into *Bsa*I sites of Cas9 expression vector, oligo primers annealing was carried out; for construction of two-gRNA-expressing vectors for gene targeting, the fragments were amplified *via* over-lapping PCR with designed primers using the vector pCBC-DT1T2 (Xing et al., 2014) as a template, and then inserted into Cas9 expression vector that was linearized by *Bsa*I through the Golden Gate cloning method.

### ARM hairy root transformation

The constructs were transformed into the *A*. *rhizogenesis* strain K599 (for soybean, cucumber, tomato, cotton, sweet potato, and tobacco) and ARqual (for *L. japonicus*) by electroporation, respectively. Composite soybean, cucumber, tomato, cotton, sweet potato, and tobacco plants were generated by one-step ARM transformation (Fan et al., 2020a; Fan et al., 2020b). Composite *L. japonicus* was generated according to the protocol (Okamoto et al., 2013). *L. japonicus* nodulation assay was performed as described by Fan et al. (2020c). *L. japonicus* composite plants with ~5 cm root lengths were inoculated with *Mesorhizobium loti* MAFF303099. For the nitrate response assay, 10 mM KNO_3_ was used and watered the transformed roots.

### Transgenic hairy roots screening, mutation type(s), and validation of genome editing

Previously, we successfully generated purple/red anthocyanin accumulation by overexpression of *AtMyb75* in transgenic *L. japonicus* hairy roots, which can be tracked as a directly visual selection marker of transgenic roots with the naked eyes in the study of rhizobia-legume symbiosis (Fan et al., 2020c). The transgenic positive hairy roots were screened by the purple/red anthocyanin accumulation on roots depending on the expression of *AtMyb75* or by visual DsRed fluorescence produced from the expression of *DsRed* reporter gene due to the different the CRISPR/Cas9 genome editing vector backbones used (**Figure 1B** and **Supplementary Figure S2**).

To analyze the mutations caused by CRISPR/Cas9, PCR/RE, (restriction enzyme) and Sanger sequencing assays were performed. Genomic DNA was extracted from independent transgenic positive hairy roots (co-transformed primary root) of 5–10 cm in length. The DNA sequences covering the CRISPR target sites of the transformed plants were amplified by PCR using gene-specific primers (**Supplementary Table S1**). *Rj7*, *GmNNL1*, *Rfg1*, *LjNLP4*, *LjSYMRK*, and *SlTRY*-specific fragments were amplified using pairs of primers Rj71/Rj72 (for *Rj7*), GmRHin1/GmRHin2 (for *GmNNL1*), GmRNco1/GmRNc4 (for *Rfg1*), LjNLP1F/LjNLP1R (for *LjNLP4*), *LjSYF*/*LjSYR* (for *LjSYMRK*), and SLTRY1/SLTRY2 (for *SlTRY*) and subsequently subjected to restriction enzyme digestion analyses and sequenced to identify the gene-edited type(s). About 10 clones for each amplicon were individually sequenced to further determine the mutation type. The experiments were replicated for three biological replicates for each transformed construct. The mean values were used for statistical analysis.

## Results

### *AtGCSpro* root expression

**Figure 2:**
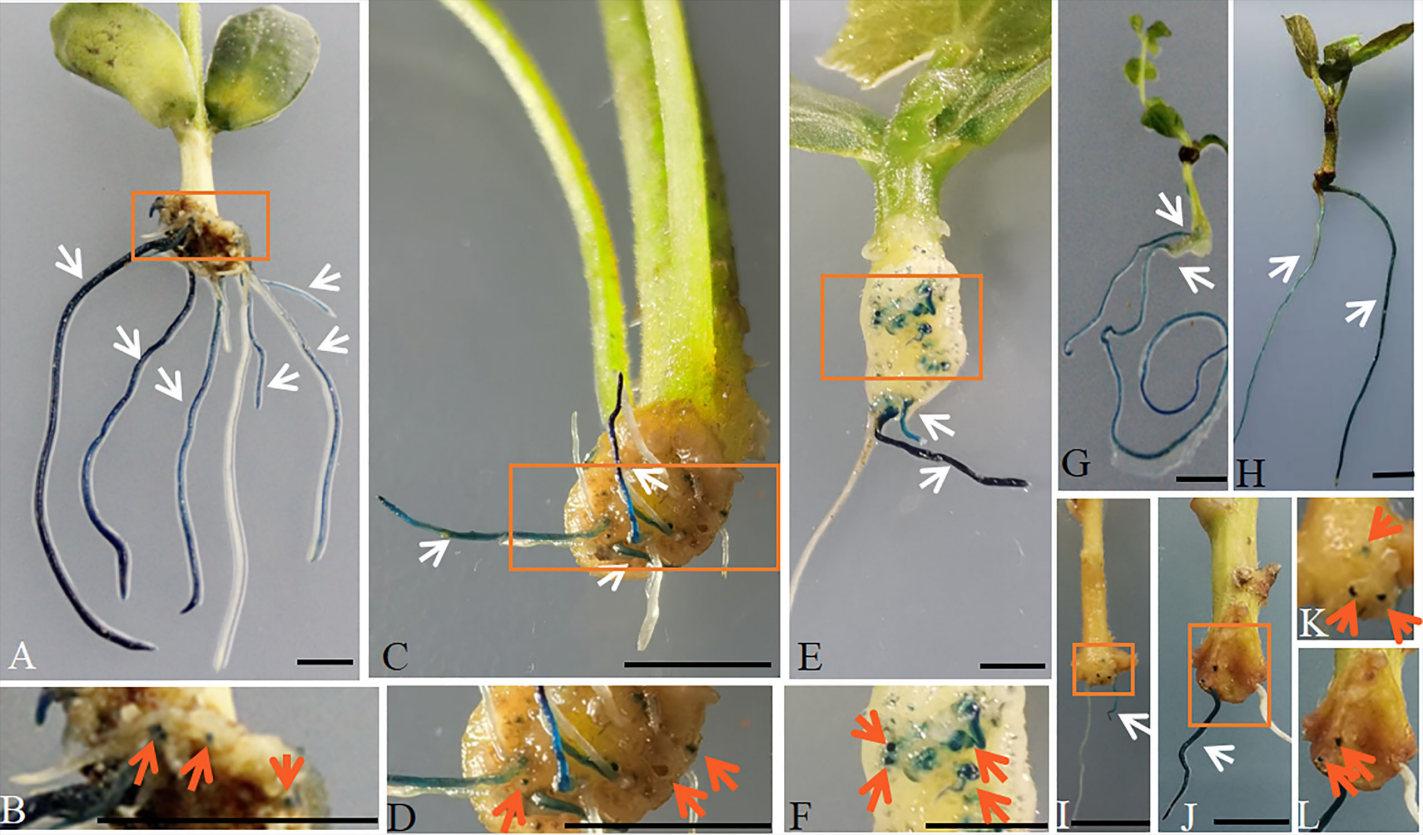
Histochemical localization of GUS activity in the p*AtGCSpro*_2411_:: *GUSPlus* hairy roots in broad dicots species by ARM transformation. High level of GUS activity was accumulated in the whole transformed p*AtGCSpro*_2411_:: *GUSPlus* roots in soybean **(A, B)**, tomato **(C, D)**, cucumber **(E, F)**, *L. japonicas* **(G)**, cotton **(H)**, tobacco **(I, K)**, sweet potato **(J, L)**, respectively. White arrows indicate the transgenic roots. Pictures **B, D, F, K, L** are closed up of sections **A, C, E, I, J** marked in the orange boxes, respectively. The GUS signal was found in the teratoma where hairy roots can emerge (indicated by orange arrows). All composite plants were observed from 16 to 21 d post-infected seedlings with K599 carrying pRedGa1 construct. Bars = 5 mm.

To assess the promoter activity of *AtGCS* at an earlier stage of the initiation of hairy roots and in developing root, a 2411-bp upstream promoter region of *AtGCS* was cloned and used to drive the expression of *GUS* (β-*Glucuronidase*). p*AtGCSpro*:: *GUSPlus* was transformed into soybean by ARM hairy roots transformation. A high level of GUS activity is found in the teratoma that is formed, from which hairy roots can emerge, and ubiquitously in the roots (**Figures 2A, B**).

In addition to experiments in soybean, we also tested the *AtGCSpro* activity in broad eudicot species, including diploid species tomato (**Figures 2C, D**), cucumber (**Figures 2E, F**), and *L*. *japonicus* (**Figure 2G**), as well as polyploid species cotton (**Figure 2H**), tobacco (**Figures 2I, K**), and sweet potato (**Figures 2J, L**). These results are in agreement with that of *AtGCSpro* in soybean (**Figures 2A, B**). Whole transformed p*AtGCSpro*::*GUSPlus* roots show a strong GUS signal in the initiation emergence regions of hairy roots and the whole roots (**Figures 2C–L**). These results indicate that *AtGCSpro* activity is broadly conserved in eudicots.

**Figure 3:**
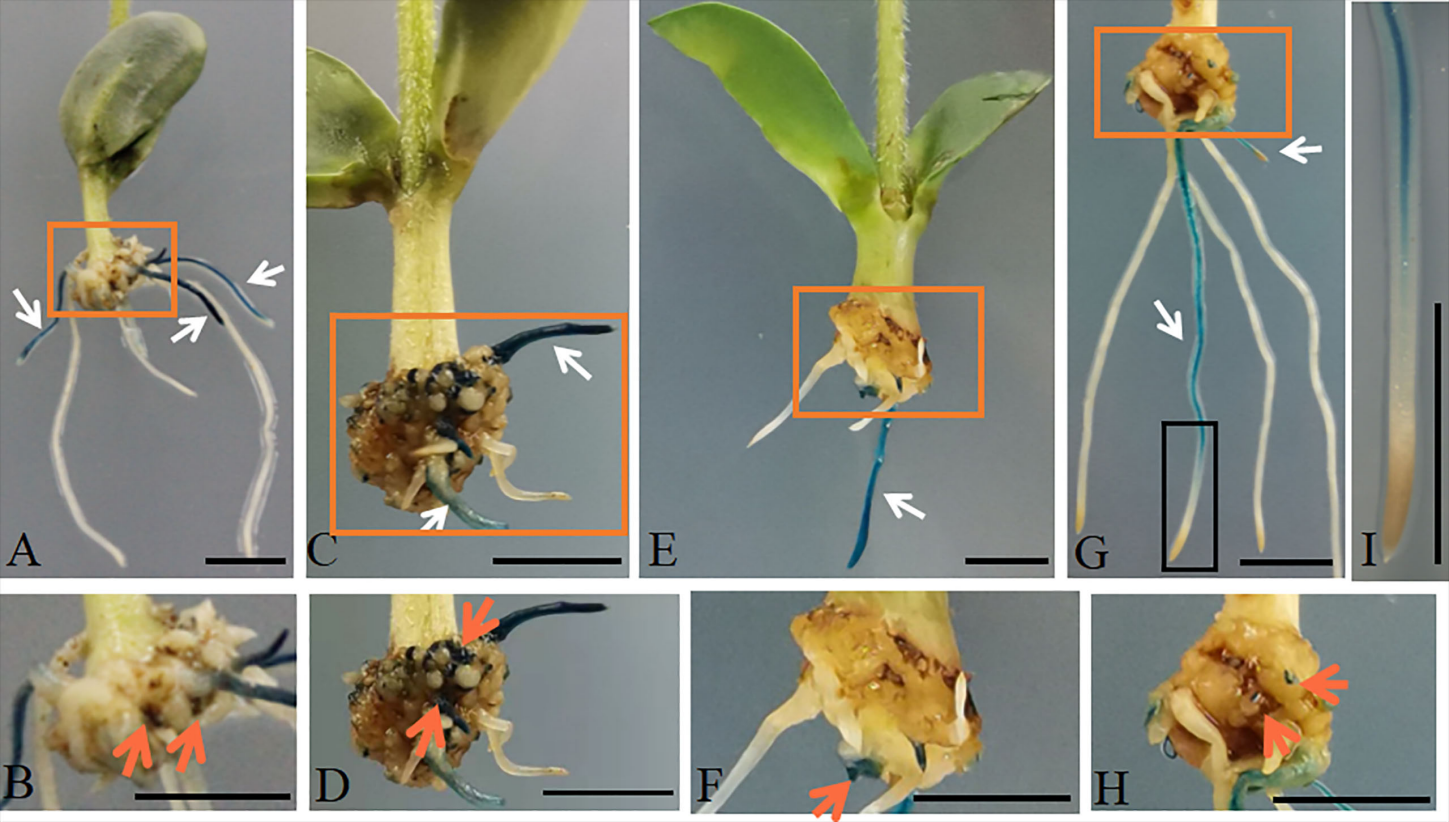
*AtGCSpro* activity assay with different shortened lengths in soybean hairy roots by ARM transformation. *AtGCSpro*_1977_:: *GUSPlus* **(A, B)**. *AtGCSpro*_1629_:: *GUSPlus* **(C, D)**. *AtGCSpro*_1178_:: *GUSPlus* **(E, F)**. *AtGCSpro*_833_:: *GUSPlus* in the soybean hairy roots **(G, H)**. The transgenic root marked in the black box in picture **(G)** is closed-up **(I)**. White arrows indicate the transgenic roots. Pictures **B, D, F,** and **H** are closed-up of sections **(A, C, E),** and **G** marked in the orange boxes, respectively. The GUS signal was found in the teratoma where hairy roots can initiate (indicated by orange arrows). All composite plants were observed from 16-day-old post-infected seedlings. Bars = 1 cm.

**Figure 4:**
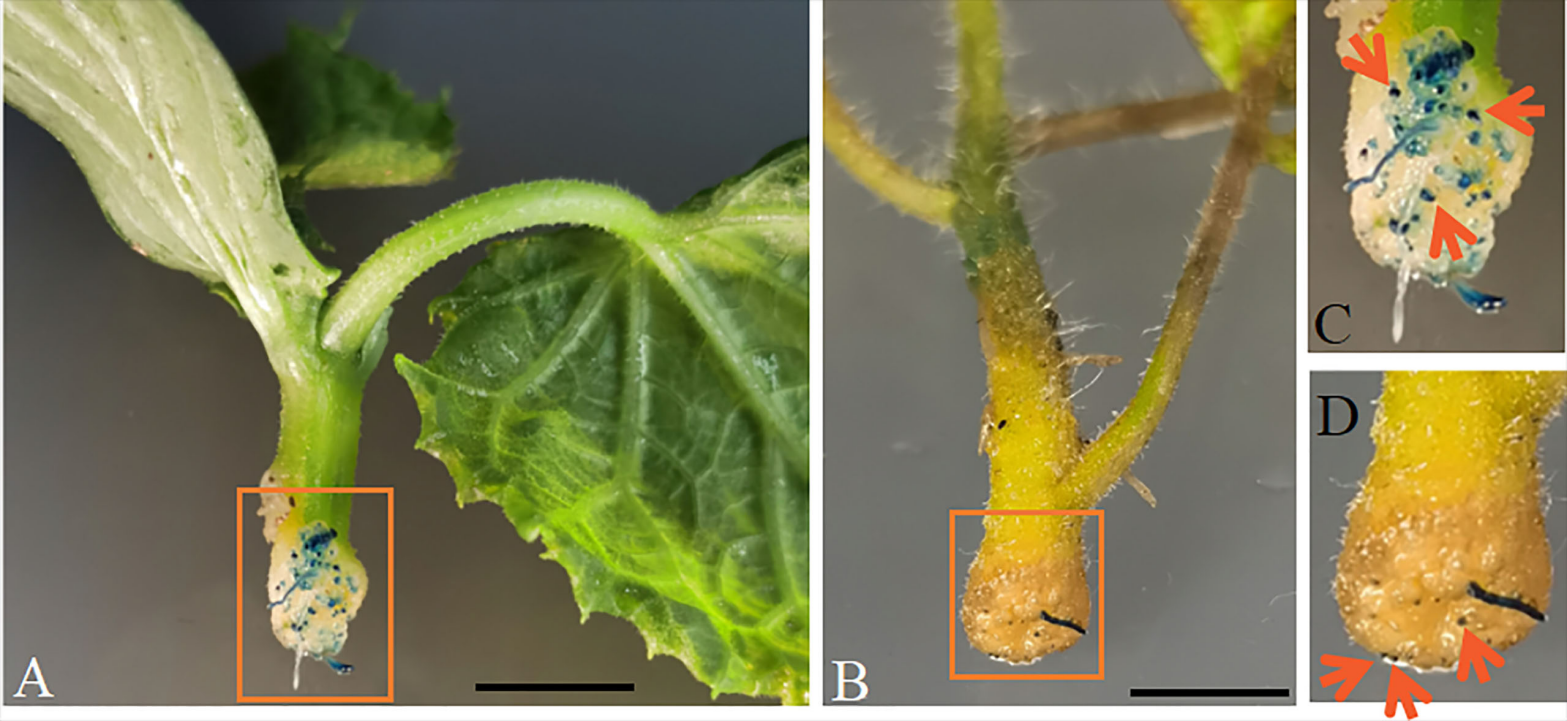
*AtGCSpro*_1178_ activity assay in other dicots hairy roots by ARM transformation. Cucumber **(A)** and tomato **(B)** transformed with *AtGCSpro*_1178_:: *GUSPlus.* GUS signal can be observed in the region where will produce transgenic roots (Shown by orange arrows in **(C and D) (C, D)**. Pictures C and D are closed-up of sections **(A, B)** marked in the orange boxes, respectively. All composite plants were observed from 16-day-old post-infected seedlings with K599 carrying p*AtGCSpro*_1178_:: *GUSPlus*. Bars = 1 cm.

To further analyze the promoter activity with different shortened lengths, truncated lengths *AtGCSpro* with 5′ deletion fragments were produced and used to drive the expression of *GUS* in soybean hairy roots. Here, we designated the full length 2411-bp sequences as *AtGCSpro*_2411_ and truncated lengths 1977-bp, 1629-bp, 1178-bp, and 833-bp sequences as *AtGCSpro*_1977_, *AtGCSpro*_1629_, *AtGCSpro*_1178_, and *AtGCSpro*_833_, respectively. There were no distinct differences in the GUS signals when comparing the transformed p*AtGCSpro*_1977_::*GUSPlus* (**Figures 3A, B**), p*AtGCSpro*_1629_::*GUSPlus* (**Figures 3C, D**), and p*AtGCSpro*_2411_::*GUSPlus* roots (**Figures 2A, B**; **Supplementary Figure S3**). In contrast, a little bit low GUS activity was observed in the transformed *AtGCSpro*_1178_::*GUSPlus* roots by histological GUS staining (**Figures 3E, F**). This result was in accordance with the relative expression analysis of *GUS* by qRT-PCR in transgenic roots, showing a little bit low expression levels of *GUS* but with no significant difference with those of *AtGCSpro*_2411_::*GUSPlus*, *AtGCSpro*_1977_::*GUSPlus*, and *AtGCSpro*_1629_::*GUSPlus* roots (**Supplementary Figure S3**). The expression level of *GUS* was the lowest in the hairy roots transformed with p*AtGCSpro*_833_::*GUSPlus* (**Figures 3G–I** and **Supplementary Figure S3**). No GUS expression is in the root tips, indicating that *AtGCSpro*_833_ has no activity in the root tip tissues (**Figures 3G, I**). In addition, we also analyzed the activity of *AtGCSpro*_1178_ in cucumber and tomato by ARM transformation (**Figures 4A–D**). The *AtGCSpro*_1178_ activity in cucumber (**Figures 4A–C**) and tomato (**Figures 4B, D**) is in agreement with those in soybean (**Figures 3E, F**). The GUS signals are strong and can be detected in the region that will develop into root meristem and in the whole developing roots.

### High H/BM induction rates in transgenic soybean and *L*. *japonicus* hairy roots using the p*AtGCSpro*-Cas9 system

**Figure 5:**
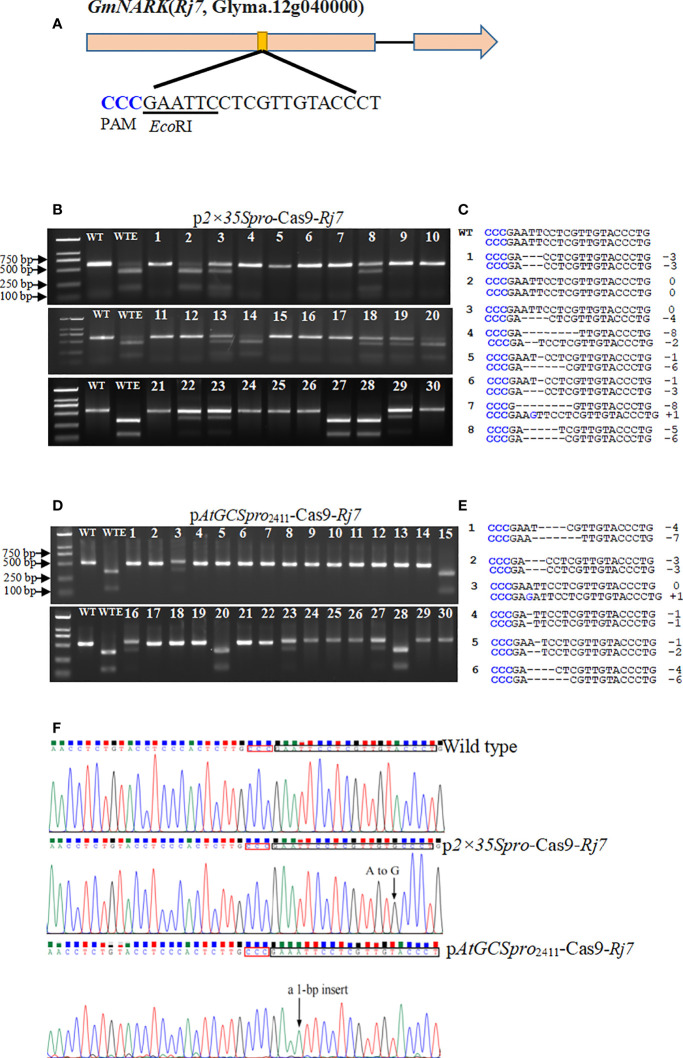
Identification of CRISPR/Cas9-induced mutation in the *GmNARK* (*Rj7*) target loci in soybean with *AtGCSpro*_2411_ and *2×35S* to drive *Cas9*, respectively. The sequence of an sgRNA designed to target a site within the first exon region of *Rj7.* The protospacer-adjacent motif (PAM) sequence is highlighted in blue and the *Eco*RI restriction site is underlined **(A)**. PCR-RE assays to detect CRISPR/Cas9-induced mutation in the *Rj7* target loci from 30 different independent p*2×35Spro*-Cas9 hairy roots **(B)**. Genotypes of eight representative mutants from transformed with p*2×35Spro*-Cas9 hairy roots identified by sequencing **(C)**. PCR-RE assays to detect CRISPR/Cas9-induced mutation in the *Rj7* target loci from 30 different independent p*AtGCSpro*_2411_-Cas9 hairy roots **(D)**. Genotypes of six representative *rj7* mutants from transformed with p*AtGCSpro*_2411_-Cas9 hairy roots identified by sequencing **(E)**. In sections B and D, Lanes WT and WTE, undigested PCR amplification fragment and digested wild-type controls by *Eco*RI, respectively. Lanes 1–30, different independent transgenic hairy roots. In sections C and E, deletions and insertions are indicated as dashes and blue letter, respectively. The types and number(s) of indels are indicated in the right column. Examples given of mutation at target site in the p*2×35Spro*-Cas9 and p*AtGCSpro*_2411_-Cas9 hairy root, respectively **(F)**. Black arrows indicate the site of indels mutation. The PAM regions and mutated target sites are shown in the black box.

Based on *AtGCSpro* activity analyses, we reasoned that *Cas9* driven by the *AtGCSpro* could be specifically transcribed in the meristematic region where the root meristem will initiate and in the whole root. To test whether *AtGCSpro* might improve H/BM frequencies in ARM transformation, we first aimed to knockout a single target site in two leguminous plant species, the important crop soybean and the model plant *L. japonicus*. The dominant traits are the most prevalent in the genome, such as, in soybean, most of characterized genes are dominant genes (Zhang et al., 2022). Due to only the transgenic roots generated H/BM at the dominant target gene site can result in loss-of-function mutation phenotype(s) in the ARM transient transformation; here, we only analyzed the H/BM-induced frequency. The soybean *GmNARK* (*Rj7*) played a crucial role in the autoregulation of nodulation (Searle et al., 2003; Lin et al., 2010) and was selected as a target site. To knock out *Rj7*, one target site in the first exon of *Rj7* containing an *Eco*RI restriction endonuclease digestion site next to the NGG region (**Figure 5A**) was selected to identify the mutation genotypes. To determine and quantify gene editing efficiency, genomic DNA was extracted from 30 independently transformed hairy roots. PCR amplicon spanning the target site was subjected to digestion by the *Eco*RI restriction enzyme, and sequenced to verify mutations-type (**Figures 5B–F** and **Table 1**). The hairy roots transformed with the p*2×35Spro*-Cas9-*Rj7* vector, 17 lines (#1, #4–7, #9–12, #15–17, #21, #24–26, and #30) among 30 independent transgenic lines are homozygous or biallelic mutations. Compared with the transgenic hairy roots transformed with the p*2×35Spro*-Cas9-*Rj7*, in the p*AtGCSpro*_2411_-Cas9-*Rj7* roots, 23 lines (#1–2, #4–14, #17–19, #21–22, #24–26, and #29–30) among 30 independent transgenic lines are homozygous or biallelic mutations (**Figure 5D**). The results indicate that the p*AtGCSpro*_2411_-Cas9 system yields 76.7% (23/30) homozygous/biallelic mutants compared with 56.7% (17/30) for the p*2×35Spro*-Cas9 system (**Figures 5B–E**; **Table 1**). *AtGCSpro*_2411_ exhibits higher efficiency than *2×35Spro* in inducing the H/BM in soybean. Various types of insertions or deletions (indels) at the target site are shown. Most of the mutation events were small indels (± 1–8 bp). No large indels (> 50 bp) were observed in 30 randomly selected sequencing mutants. Noticeably, some H/BM mutants with 3n indels (**Figures 5C, E** and **Supplementary Figure S4**).

**Table 1:** Comparison of the targeted mutation efficiency in transgenic soybean hairy roots by ARM transformation using different truncated promoters to drive the expression of *Cas9*.

Cas9 system	No. of H/BM roots/ no. of roots examined	H/BM rate (%)
p*2×35Spro*-Cas9-*Rj7*	17/30	56.7%
p*AtGCSpro*_2411_-Cas9-*Rj7*	23/30	76.7%
p*AtGCSpro*_1178_-Cas9-*Rj7*	21/30	70.0%
p*AtGCSpro*_833_-Cas9-*Rj7*	10/20	50.0%

To determine the shortest *AtGCSpro* length used to drive the expression of *Cas9* without sacrificing H/BM efficiency, we also generated p*AtGCSpro*_1178_-Cas9 and p*AtGCSpro*_833_-Cas9 systems to knockout *Rj7.* There was a slightly decreased H/BMs efficiency by p*AtGCSpro*_1178_-Cas9 (21/30; 70%) compared with p*AtGCSpro*_2411_-Cas9 (76.7%) (**Supplementary Figures S5A**, **Figure 5D**, and **Table 1**). However, the p*AtGCSpro*_833_-Cas9 system substantially affects the genome editing efficiency of H/BM at a rate of 50% (10/20) compared with 76.7% (23/30) for the p*AtGCSpro*_2411_-Cas9 system, which is even less than the p*2×35Spro*-Cas9 system (**Supplementary Figures S5A–C**, **Figure 5D**, and **Table 1**). Therefore, to minimize the construct’s size, for genome editing, we recommend using p*AtGCSpro*_1178_-Cas9 system.

**Figure 6:**
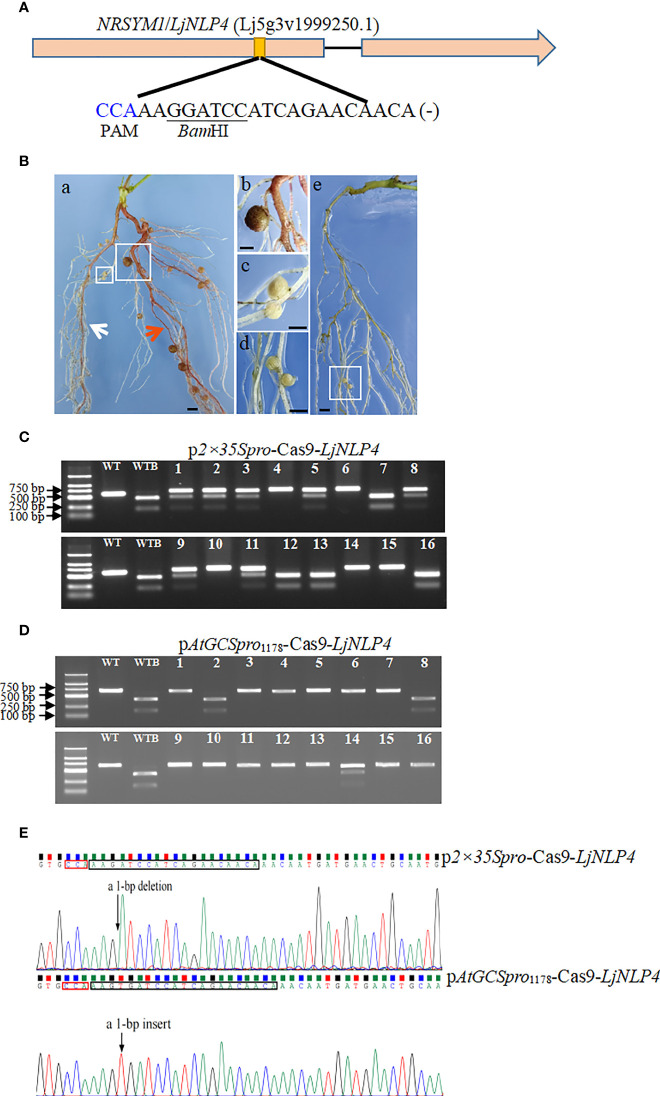
PCR-RE assay mutation efficiency in the *NRSYM1*/*LjNLP4* target loci in *L*. *japonicus*. Sequence of an sgRNA designed to target a site within the first exon region of *LjNLP4.* The PAM sequence is highlighted in blue and the *Bam*HI restriction site is underlined **(A)**. *L*. *japonicus* hairy roots with edited *NRSYM1*/*LjNLP4* allele. Transgenic hairy roots with edited *LjNLP4Ljnlp4* (white arrow indicated), *Ljnlp4Ljnlp4* allele (red arrow indicated) in picture a, and the wild type in picture e; pictures b, c, and d are closed-up of sections a (big white box), a (small white box) and e marked in the boxes, respectively. Bars = 1 mm **(B)**. PCR-RE assays to detect mutation efficiency in the *NRSYM1*/*LjNLP4* target loci. Lanes 1–16, different independent transgenic hairy roots. Lanes WT and WTB, undigested PCR amplification fragment and digested wild-type controls by *Bam*HI, respectively **(C, D)**. Five lines (#4, #6, #10, #14, and #15) were homozygous or biallelic mutations **(C)**. 13 lines (#1, #3-7, #9-13, #15, and #16) were homozygous or biallelic mutations **(D)**. An example shown of mutation in the target site in p*2×35Spro*-*Cas9* and p*AtGCSpro*_1178_-*Cas9* hairy root, respectively **(E)**.

In *L. japonicus*, the *NRSYM1*/*LjNLP4* (Lj5g3v1999250.1) functions as a master regulator for nitrate-dependent symbiotic gene expression and inhibits nodulation when surplus nitrate is in soil. *LjNLP4* was selected as the targeted gene because mutations in *LjNLP4* result in conveniently observable “nodule” phenotypes, such as defects in high nitrate concentrations. The *ljnlp4* mutant can form mature nitrogen-fixing nodules in the presence of a high nitrate concentration (Suzuki et al., 2013; Nishida and Suzaki, 2018; Nishida et al., 2021). One sgRNA was designed to target the *LjNLP4* in *L*. *japonicus* (**Figure 6A**). To estimate the H/BM rate for *ljnlp4*, as the criterion of success, we used whether leghemoglobin-rich pink mature nodules formed on transgenic hairy roots in the presence of a high nitrate concentration. Using this classification, 40 transgenic plants were analyzed, and 33 of them (82.5%) were independent transgenic hairy roots lines transformed with the p*AtGCSpro*_1178_-Cas9-*LjNLP4* showing mature nodules in the presence of high nitrate concentrations, compared with 32.5% (13/40) for p*2×35Spro*_1178_-Cas9-*LjNLP4* (**Figure 6B**). To further accurately determine the gene-editing efficiency, PCR amplicons amplified from each independent transgenic hairy root covering the target site were subjected to digestion by restriction enzyme *Bam*HI and Sanger sequencing. Sixteen independent transgenic roots were tested. The *LjNLP4* targeted site was successfully edited using the p*2×35Spro*-Cas9 and p*AtGCSpro*_1178_-Cas9 systems (**Figures 6C–E**). However, the editing efficiencies are distinct between the p*2×35Spro*-*Cas9*-*LjNLP4* and p*AtGCSpro*_1178_-Cas9-*LjNLP4* systems (**Figures 6C–E** and **Table 2**). The H/BM rate for *ljnlp4* was 31.3% (5/16) for the p*2×35Spro*-Cas9 system; much lower than the 81.3% (13/16) achieved using the p*AtGCSpro*-Cas9 system (**Figures 6B–D** and **Table 2**). Based on the results and previous mutation frequency results in soybean, on average, p*AtGCSpro*_1178_-Cas9 system shows a 1.7 times higher H/BM frequency than the p*2×35Spro*-Cas9 system for a single target site in the genome (proportion of H/BM-induced frequency using p*AtGCSpro*_1178_-Cas9 system compared with that of the p*2×35Spro*-Cas9 system in soybean and *L. japonicus*).

**Table 2:** Comparison of p*AtGCSpro*_1178_-Cas9*-LjNLP4* and p*2×35Spro*-Cas9*-LjNLP4* genome editing efficiency in *L*. *japonicus* hairy roots.

Cas9 system	No. of H/BM roots/ no. of roots examined	H/BM rate (%)
p*2×35Spro*-Cas9-*LjNLP4*	5/16	31.3%
p*AtGCSpro*_1178_-Cas9-*LjNLP4*	13/16	81.3%

Besides *AtGCSpro*, we also evaluated the *ubiquitin* promoter (Ma et al., 2015) and *YAO* promoter (a high-efficiency germ cell-specific promoter in *Agrobacterium*-mediated genetic transformation and with high activity in roots) (Li et al., 2010; Yan et al., 2015; Feng et al., 2018; Fan et al., 2020a; Fan et al., 2020b). The results indicate that *AtGCSpro* is the most efficient promoter for inducing H/BM, outperforming the *ubiquitin*, *YAO*, and *CaMV 35S* promoters in both transgenic soybean and *L*. *japonicus* hairy roots (**Tables S2 and S3**).

### Using the p*AtGCSpro*-Cas9 system for simultaneously targeting multiple genome loci(s) in ARM transformation

In plants, multiple genomic sites need to be edited simultaneously, resulting in the observable phenotype(s), such as studying multiple functionally related genes or the knockout of functionally redundant genes (Ma et al., 2016). Due to high-efficiency H/BM induction rates for a single target site using the *AtGCSpro*-Cas9 system relative to other systems, we next assessed the efficiency of p*AtGCSpro*_1178_-Cas9-induced H/BMs when simultaneously targeting two genome sites in soybean, *L*. *japonicus*, and tomato. In soybean, which is resistant to nodulation, *GmNNL1* (Glyma.02g076900) (Zhang et al., 2021) and *Rfg1* gene (Fan et al., 2017) were targeted simultaneously. p*2×35Spro*-Cas9-*GmNNL1Rfg1* and p*AtGCSpro*_1178_-Cas9-*GmNNL1Rfg1* achieved H/BM frequencies of 0% (0/30) and 6.7% (2/30) at the *GmNNL1* site, 33.3% (10/30) and 83.3% (25/30) at the *Rfg1* site, respectively (**Supplementary Figures S6A–H** and **Table 3**). The H/BM frequencies for *gmnnl1gmnnl1rfg1rfg1* were 0% (0/30) (p*2×35Spro*-Cas9-*GmNNL1Rfg1*) and 6.7% (2/30) (p*AtGCSpro*_1178_-Cas9-*GmNNL1Rfg1*), respectively (**Supplementary Figures S6A–H** and **Table 3**).

**Table 3:** Comparison of p*AtGCSpro*_1178_-Cas9*-GmNNL1Rfg1* and p*2×35Spro*-Cas9*-GmNNL1Rfg1* genome editing efficiency in soybean hairy roots.

Cas9 system	*gmnnl1gmnnl1* H/BM rate at *GmNNL1* site (no. of roots with H/BM at *GmNNL1* site/no. of roots examined)	*rfg1rfg1* H/BM rate at *Rfg1* site (no. of roots with H/BM at *Rfg1* site/no. of roots examined)	*gmnnl1gmnnl1*/*rfg1rfg1*H/BM rate at both *GmNNL1* and *Rfg1* sites (no. of roots with H/BM at both *GmNNL1* and *Rfg1* sites/no. of roots examined)
p*2×35Spro*-Cas9-*GmNNL1Rfg1*	0% (0/30)	33.3% (10/30)	0% (0/30)
p*AtGCSpro*_1178_-Cas9-*GmNNL1Rfg1*	6.7% (2/30)	83.3% (25/30)	6.7% (2/30)

In *L*. *japonicus*, two genomic target sites were analyzed, *LjNLP4* (Suzuki et al., 2013; Nishida and Suzaki, 2018; Nishida et al., 2021) and *LjSYMRK* (Wang et al., 2016). Compared with p*2×35Spro*-Cas9-*LjNLP4LjSYMRK*, H/BM frequencies of the two *LjNLP4* and *LjSYMRK* target sites were significantly increased from 30.0% (9/30) to 83.3% (25/30) at the *LjNLP4* site, from 26.7% (8/30) to 83.3% (25/30) at the *LjSYMRK* site, when using p*AtGCSpro*_1178_-*Cas9*-*LjNLP4LjSYMRK*. The H/BM frequencies for *ljnlp4ljnlp4ljsymrkljsymrk* were 10.0% (3/30) (p*2×35Spro*-*Cas9*-*LjNLP4LjSYMRK*) and 66.7% (20/30) (p*AtGCSpro*_1178_-*Cas9*-*LjNLP4LjSYMRK*), respectively (**Supplementary Figures S7A–G** and **Table 4**).

**Table 4:** Comparison of p*AtGCSpro*_1178_-Cas9*-LjNLP4LjSYMRK* and p2×*35Spro*-Cas9*-LjNLP4LjSYMRK* genome editing efficiency in *L*. *japonicus* hairy roots.

Cas9 system	*ljnlp4ljnlp4* H/BM rate at *LjNLP4* site (no. of roots with H/BM at *LjNLP4* site/no. of roots examined)	*Ljsymrkljsymrk* H/BM rate at *LjSYMRK* site (no. of roots with H/BM at *LjSYMRK* site/no. of roots examined)	*ljnlp4ljnlp4*/*ljsymrkljsymrk* H/BM rate at both *LjNLP4* and *LjSYMRK* sites (no. of roots with H/BM at both *GmNNL1* and *Rfg1* sites/no. of roots examined)
p*2×35Spro*-Cas9-*LjNLP4LjSYMRK*	30.0% (9/30)	26.7% (8/30)	10.0% (3/30)
p*AtGCSpro*_1178_-Cas9-*LjNLP4LjSYMRK*	83.3% (25/30)	83.3% (25/30)	66.7% (20/30)

In tomato (*Solanum lycopersicum*), two gRNAs were designed to introduce mutations into the tomato endogenous gene *TRYPTICHON* (*SlTRY*, Solyc01g095640.1.1) (Tominaga-Wada et al., 2013). Consistent with these previous observations, using the *AtGCSpro*_1178_ promoter to direct *Cas9* expression can lead to a higher H/BM induction rates. At the sgRNA1 target site, p*2×35Spro*-Cas9-*SlTRY* and p*AtGCSpro*_1178_-Cas9-*SlTRY* result in H/BM frequencies of 23.3% (7/30) and 91.3% (21/23), and at the sgRNA2 target site, 13.3% (4/30) and 78.3% (18/23), respectively. The H/BM induction rates for the two simultaneously targeted sites were 6.7% (2/30) (p*2×35Spro*-Cas9-*SlTRY*) and 65.2% (15/23) (p*AtGCSpro*_1178_-Cas9-*SlTRY*), respectively (**Supplementary Figures S8A–D** and **Table 5**).

**Table 5:** Comparison of p*AtGCSpro*_1178_-Cas9*-SlTRY* and p2×*35Spro*-Cas9*-SlTRY* genome editing efficiency in tomato hairy roots.

Cas9 system	H/BM rate at target site 1 (no. of roots with H/BM at target site 1/no. of roots examined)	H/BM rate at target site 2 (no. of roots with H/BM at target site 2/no. of roots examined)	H/BM rate at both target sites 1 and 2 (no. of roots with H/BM at both target site 1 and 2/no. of roots examined)
p*2×35Spro*-Cas9- *SlTRY*	23.3% (7/30)	13.3% (4/30)	6.7% (2/30)
p*AtGCSpro*_1178_-Cas9-*SlTRY*	91.3% (21/23)	78.3% (18/23)	65.2% (15/23)

Based on the above results, the p*AtGCSpro*-Cas9 system always substantially enhances the H/BM-induced frequency over the p*2×35Spro*-Cas9 system in soybean, *L*. *japonicus*, and tomato. By using the p*AtGCSpro*-Cas9 system, we achieved an average H/BM frequency 8.3-fold (proportion of H/BM-induced frequency using p*AtGCSpro*_1178_-Cas9 system compared with that of the p*2×35Spro*-Cas9 system in soybean, *L. japonicus* and tomato) higher than the p*2×35Spro*-Cas9 system for two targeted site(s) in the genome.

## Discussion

### The p*AtGCSpro*-Cas9 system significantly improves H/BM efficiencies relative to the p*2×35Spro*-Cas9 system by ARM transformation

In *Arabidopsis*, *AtGCS* encodes the first enzyme of glutathione (GSH; 7-glutamylcys teinyl glycine) biosynthesis, γ-glutamylcysteine synthetase (7-GCS; EC 6.3.2.2; May and Leaver, 1994). *AtGCSpro* is involved in the control of mitosis cell cycle during the G1 to S phase and regulates the initiation and maintenance of cell division in the root apex (Vernoux et al., 2000). However, the promoter activity of *AtGCS* has not been reported. In this study, our results indicated that *AtGCSpro* had a high activity in the initiation emergence regions of hairy roots, later, in the root meristem, and in the developing roots. The p*AtGCSpro*-Cas9 system markedly improves H/BM efficiencies relative to the p*2×35Spro*-Cas9 system in soybean, *L. japonicus*, and tomato by ARM transformation. Combined the expression of *AtGCSpro* in this study with previous studies on the functions of that in specific cell cycle (Cheng et al., 1995; Vernoux et al., 2000), we reasoned that large numbers of Cas9-driven by *AtGCSpro* expressed in the roots during the G1-to-S phase and loosened chromatin DNA structure and single-strand DNA condition contribute Cas9’s cutting of the DNA strands to generate a DNA-strand break. In particular, single-strand chromatin DNA is subjected to cutting and is introduced to the mutations, which will result in homozygous mutants following cell mitosis cycles. Therefore, it is reasonable that the *AtGCSpro*-Cas9 system can produce higher H/BM rates than p*2×35Spro*-Cas9.

Previous studies have indicated that using the *YAO* promoter to drive *Cas9* expression in CRISPR/Cas9 constructs leads to high-efficiency genome editing in *Arabidopsis* by *A. tumefaciens*–mediated genetic transformation (Yan et al., 2015; Feng et al., 2018). In contrast, in this study, the *YAO* promoter showed a much lower efficiency than the *2×35S* promoter in driving Cas9 expression in soybean and *L. japonicus* roots mediated by ARM transformation (**Supplementary Tables S2 and S3**), despite high *YAO* promoter activity in roots (Fan et al., 2020a; Fan et al., 2020b). This might be because of the target of the T-DNA in ARM transformation but not the germline cells in *A*. *tumefaciens*–mediated stable genetic transformation in *Arabidopsis*. Additionally, *AtGCSpro* is higher efficient promoter in inducing H/BM, outperforming the constitutive expression promoter *ubiquitin* in both transgenic soybean and *L*. *japonicus* hairy roots (**Supplementary Tables S2 and S3**). Based on these results, we concluded that *Cas9* expression timing and tissue specificity are crucial to the editing efficiency of the CRISPR/Cas9 system in ARM transformation.

### The *pAtGCSpro-*Cas9 system indicates great potential in multiplex genome editing

Gene duplications are especially prevalent in plants, and the genomes of most extant angiosperm species result from a series of segmental or whole-genome duplication events. At least 70% of all angiosperms underwent at least one episode of polyploidization in their evolutionary history (Leitch and Bennett, 1997; Comai, 2000; Soltis and Soltis, 2000; Wendel, 2000; Qiao et al., 2019). Some species have undergone multiple occurrences of polyploidization in the coding portions of the genome, which are organized hierarchically into families or superfamilies. More than 50% of genes belong to gene family members in eukaryotes (Chervitz et al., 1998; Koonin et al., 1998; Semple and Wolfe, 1999; Thornton and DeSalle, 2000; Blanc and Wolfe, 2004; Xu et al., 2022). Many agriculturally important crops are polyploid plants, such as tetraploid potato (*Solanum tuberosum*), oilseed rape (*Brassica napus*), tobacco, cotton (*Gossypium* spp.), hexaploid bread wheat, sweet potato, and octoploid strawberry (*Fragaria × ananassa*) (Yang et al., 2017; Abe et al., 2019; Edger et al., 2019; Gao, 2021). To analyze the mutant phenotype(s), these duplicated genes with redundant functions must be simultaneously mutated to generate homozygous/multi-allelic changes for dominant target genes at alleles site. Using the p*AtGCSpro*-Cas9 system, the average H/BM frequency is 8.3-fold higher than the p*2×35Spro*-Cas9 system for two simultaneously targeted sites in the genome. Compared with targeting a single genomic site (1.7-fold increased), the efficiency of simultaneous homozygous/biallelic mutagenesis in a single event is significantly increased for targeting two genomic sites using the p*AtGCSpro*-Cas9 system. Therefore, with the increasing of genomic targeted sites, the H/BM frequency is more significantly increased using the p*AtGCSpro*-Cas9 system. The p*AtGCSpro*-Cas9 system provides a powerful tool for analyzing the loss-of-function phenotypes of duplicated genes in the diploid and polyploids plants for multiple genomic targeted editing.

In the traditional genetic transformation mediated by *Agrobacterium*, generating recessive change at multiple target sites is also very important. Although homozygous mutants can also be obtained from heterozygotes mutants at the sgRNA target site by plant self-crossing, a longer experiment was required, and it was laborious to screen and identify the homozygous mutants. Previous research has indicated that regeneration plants using the root or root tip as explants had been reported in some plants, such as *L*. *japonicus* (Lombari et al., 2003), tomato (Peres et al., 2001), Chicory (Matvieieva et al., 2011), and *Medicago truncatula* (Iantcheva et al., 2005). This suggests that the transgenic hairy roots with H/BM at multiple targeted sites could be used as explants to induce the regeneration plants in some plants. Bernard et al. (2019) reported that the edited hairy roots can be used for explants to generate the whole transgenic plant in chicory. The p*AtGCSpro*-Cas9 system is a greatly convenient for plant genetic engineering breeding involving the simultaneous alteration of multiple homoeologs with H/BM in the transformation of T_0_ generation. This is a promising technical breakthrough for accelerating plant breeding for simultaneous H/BM at multiple genome target sites to eliminate “deleterious” genes with establishing regeneration plants using the root or root tip as explants in some plant species. The p*AtGCSpro*-Cas9 system would propel plant breeding and accelerate the generation of homologous mutants with multiplexed genome modifications of homologous genes or gene families in a much shorter time than conventional breeding techniques. Additionally, the genotyping screening of H/BM will greatly reduce the working burden at multiple sites.

### The conserved activity of *AtGCSpro* in eudicots species suggests that the *AtGCSpro-*Cas9 system might have much potential for generating high frequency H/BMs in a wide range of dicots plant species in ARM transformation

In this study, the *AtGCSpro-*Cas9 system always indicates notably increased homozygous/biallelic targeted mutation efficiency in selected species soybean, *L. japonicus*, and tomato tested than the p*2×35S_pro_*-Cas9 system, although the rates of H/BM-induced are different in different species at different target sites. Furthermore, *AtGCSpro* indicates a strong activity in broad eudicots species, such as soybean, tomato, cucumber, *L. japonicus*, tobacco, sweet potato, and cotton. The conserved activity of *AtGCSpro* in eudicots species suggests that the *AtGCSpro-*Cas9 system might induce higher H/BM in a wide range of dicots plant species in ARM transformation.

In this study, as expected, in the H/BM-induced mutants, we found that some homozygous/biallelic mutants with 3n indels at target site. As protein coding genes are read in units of three (codons), the 3n indels would result in only insert or delete 1 or several amino acids in the corresponding coding protein, and some homozygous/biallelic mutants with 3n indels (such as indels of 3bp, 6bp, 9bp, …) may not be loss-of-function mutants. The generated homozygous/biallelic mutants with non-3n indels at the dominant target gene site are required because they can produce complete loss-of function mutants. Therefore, it is crucial to establish a high-efficiency CRISPR/Cas9 system with higher H/BM mutation efficiency applied for ARM transformation because a certain ratios 3n indels mutants (randomly generated) are produced in the homozygous/biallelic mutants. Besides the *AtGCS* promoter-driven CRISPR/Cas9, in the future, H/BM efficiency may be improved by optimizing the *AtGCSpro-*Cas9 system, such as using plant endogenous *GCS* gene promoter to drive the *Cas9* expression, endogenous U6 promoter-driven sgRNA, codon-optimized *Cas9*, tRNA for multiplexing, a modified sgRNA scaffold, and intronized *Cas9* (Li et al., 2014; Dang et al., 2015; Xie et al., 2015; Grützner et al., 2020; Huang et al., 2022). For example, in soybean, the genome editing efficiency was increased by 1.8-fold to 6.3-fold when the *GmU6-10* promoter drove the sgRNA expression by replacing the *Arabidopsis AtU6-26* gene promoter with CRISPR/Cas9 (Sun et al., 2015). This study shows that the *AtGCSpro*-Cas9 system is a viable tool for use in inducing H/BMs in a wide scope of plant species in the ARM transformation.

## Data availability statement

The original contributions presented in the study are included in the article/**Supplementary Material**. Further inquiries can be directed to the corresponding authors.

## Author contributions

YF and SHL conceived and designed the experiments, and wrote the paper. SL, XW, QL, WP, ZZ, PC, and SG performed the work and analyzed data. All authors contributed to the article and approved the submitted version.

## Funding

This work was supported by National Natural Science Foundation of China (no. 31271751), and partially funded by Natural Science Foundation of Shandong province (no. ZR2012CL16 and ZR2010CQ025) and Industrial Upgrading Project of Shandong Agricultural Science and Technology Park (2019YQ035).

## Acknowledgments

We thank Prof. Zhe Yan (Northeast Institute of Geography and Agroecology, CAS, China) for kindly providing the *L. japonicas* seeds and strains *Mesorhizobium loti* MAFF303099, Prof. Yaoguang Liu and Letian Chen (South China Agricultural University) for pYLsgRNA-AtU3d and pYLCRISPR/Cas9Pubi-B, and Prof. Qijun Chen (China Agricultural University) for pHSE401.

## Conflict of interest

The authors declare that the research was conducted in the absence of any commercial or financial relationships that could be construed as a potential conflict of interest.

## Publisher’s note

All claims expressed in this article are solely those of the authors and do not necessarily represent those of their affiliated organizations, or those of the publisher, the editors and the reviewers. Any product that may be evaluated in this article, or claim that may be made by its manufacturer, is not guaranteed or endorsed by the publisher.

## Supplementary material

Figure S1 The schematic diagrams of the pRSE401.

Figure S2 *DsRed* and *AtMyb75* as visual reporter genes used for screening transgenic hairy roots in soybean, tomato, and *L. japonicus*, respectively. Hairy roots were induced from soybean **(A-B)**, tomato **(C-D)**, and *L. japonicas* **(E-G)**, respectively. Sections A, C, E, and G were pictured under the bright light. DsRed fluorescence was observed in transgenic positive soybean **(B)**, tomato **(D)**, and *L. japonicus* **(F)** hairy roots, and purple/red anthocyannin in *L. japonicus* **(G)**, respectively. Arrows indicated transgenic positive hairy roots. Bars=5 mm.

Figure S3 Relative expression levels of GUS in the transformed with pRedGa1/2/3/4 soybean hairy roots by a qRT-PCR assay. Data were analyzed using Microsoft office Excel 2016 and Data Processing System (DPS) statistical software. The averages and standard deviations were calculated. ** represented very significantly different at the p=0.01 value given.

Figure S4 Sanger sequencing chromatogram of analysis on the H/BM mutations at 15 *Rj7* target site mediated by p*AtGCSpro*_2411_-Cas9-*Rj7* system were given.

Figure S5 Mutation at *Rj7* target site mediated by p*AtGCSpro*_1178_-Cas9 and p*AtGCSpro*_833_-Cas9 system in soybean, respectively, by PCR-RE assays and Sanger sequencing analysis. PCR-RE assays mutation efficiency in the *Rj7* target loci from different independent p*AtGCSpro*_1178_-Cas9 and p*AtGCSpro*_833_-Cas9 hairy roots, respectively *(A, B)*. Lanes WT and WTE, undigested PCR amplification fragment and digested wild-type controls by *Eco*RI, respectively. Lanes 1-30, different independent transgenic hairy roots **(A)**. Lane 1, 3-11, 13, 17-18, 20, 22-23, 25-28, and 30 were homozygous/ biallelic mutations **(A)**. Lanes 1-20, different independent transgenic hairy roots **(B)**. Lane 1, 3, 5-7, 12, 14, and 17-19 were homozygous/ biallelic mutations **(B)**. An example of sequencing analysis on the mutation at *Rj7* target site was given in p*AtGCSpro*_1178_-Cas9 and pAtGCSpro833-Cas9 system, respectively. Black arrows indicate the site of indels mutation. The PAM region and mutated target site are shown in the box **(C)**.

Figure S6 Mutation at *GmNNL1Rfg1* target sites in soybean mediated by p*2×35Spro*-Cas9 and p*AtGCSpro*_1178_-Cas9 system, respectively, and PCR-RE assays and Sanger sequencing analysis. Sequence of an sgRNA designed to target a site within the fifth exon region of *GmNNL1* **(A)** and *Rfg1* **(B)**, respectively. The *Hind*III **(A)** and *Nco*I **(B)** restriction site is underlined, respectively. The PAM sequence is highlighted in blue. PCR-RE analysis the targeted mutation at *GmNNL1* site using *Hind*III restriction enzyme digestion. In the hairy roots transformed with the p2*×35Spro*-Cas9-*GmNNL1Rfg1* vector, no homozygous or biallelic mutations lines were obtained among 30 independent transgenic lines **(C)**. PCR-RE analysis the targeted mutation at *Rfg1* site using *Nco*I restriction digestion. In the hairy roots transformed with the p*2×35Spro*-Cas9-*GmNNL1Rfg1* vector, 41 10 lines (#5-7, #11, #14-17, #19, and #26) were homozygous or biallelic mutations among 30 independent transgenic lines **(D)**. PCR-RE analysis the targeted mutation in *GmNNL1* site in the hairy roots transformed with the p*AtGCSpro*_1178_-Cas9-*GmNNL1Rfg1* vector using *Hind*III restriction enzyme digestion. 2 lines (#28, and #30) were homozygous or biallelic mutations among independent transgenic lines **(E)**. PCR-RE analysis the targeted mutation at *Rfg1* site in the hairy roots transformed with the p*AtGCSpro*_1178_-Cas9-*GmNNL1Rfg1* vector using *Nco*I restriction enzyme digestion. 25 lines (#1-4, #7-11, #13-15, #17-28, and #30 ) were homozygous or biallelic mutations among 30 independent transgenic lines **(F)**. An example of sequencing analysis on the mutation at *GmNNL1* target site was given in p*AtGCSpro*_1178_-Cas9-*GmNNL1Rfg1* and p*2×35Spro*-Cas9-*GmNNL1Rfg1* system, respectively. WT represents wild type. Black arrows indicate the site of indels mutation **(G)**. An example of sequencing analysis on the mutation at *Rfg1* target site was given in p*AtGCSpro*_1178_-Cas9-*GmNNL1Rfg1* and p*2×35Spro*-Cas9-*GmNNL1Rfg1* system, respectively. Black arrows indicate the site of indels mutation *(H)*.

Figure S7 Mutation at *LjNLP4LjSYMRK* target sites in *L. japonicus* mediated by p*2×35Spro*-Cas9 and p*AtGCSpro*_1178_-Cas9 system, respectively, and PCR-RE assays and Sanger sequencing analysis. Sequence of an sgRNA designed to target a site within the first exon region of *LjNLP4* **(A)** and within the thirteenth exon region of *LjSYMRK* **(B)**, respectively. The *Bam*HI **(A)** and *Pst*I **(B)** restriction site is underlined, respectively. The protospaceradjacent motif (PAM) sequence is highlighted in blue. PCR-RE assays to detect CRISPR/Cas9-induced mutation in the *LjNLP4* target loci using *Bam*HI from 30 different independent p*2×35Spro*-Cas9 *LjNLP4LjSYMRK* **(C)** and p*AtGCSpro*_1178_-Cas9-*LjNLP4LjSYMRK* **(D)** hairy roots, respectively. 9 lines (#3, #4, #9, #10, #12, #17, #21, #24, and #29) were homozygous or biallelic mutations **(C)**. 25 lines (#2-7, #9-15, #17-24, #26-28, and #30) were homozygous or biallelic mutations **(D)**. PCR-RE assays to detect CRISPR/Cas9-induced mutation in the *LjSYMRK* target loci using *Pst*I from 30 different independent p*2×35Spro*-Cas9-*LjNLP4LjSYMRK* **(E)** and p*AtGCSpro*_1178_-Cas9-*LjNLP4LjSYMRK* **(F)** hairy roots, respectively. 8 lines (#1, #4, #8, #12, #19, #22, #24, and #28) were homozygous or biallelic mutations *(E)*. 25 lines (#1-6, #8-14, #16, #18-20, and #22-29) were homozygous or biallelic mutations **(F)**. Several examples of sequencing analysis on the mutation at *LjSYMRK* target site was given in p*2×35Spro*-Cas9 and p*AtGCSpro*_1178_-Cas9 system, respectively **(G)**.

Figure S8 Mutation at SlTRY target sites in tomato mediated by p*2×35Spro*-Cas9 and p*AtGCSpro*_1178_-Cas9 system, respectively, and PCR-RE assays and Sanger sequencing analysis. Sequences of two sgRNA designed to target sites within the first and second exons region of *SlTRY*. The PAM sequence is highlighted in blue and the *Eco*RI restriction site is underlined **(A)**. PCR-RE analysis the targeted mutation at *SlTRY* site using *Eco*RI restriction enzyme digestion. In the hairy roots transformed with the p*2×35Spro*-Cas9-*SlTRY* vector **(B)**. PCR-RE analysis the targeted mutation at *SlTRY* site using *Eco*RI restriction digestion. In the hairy roots transformedwith the p*AtGCSpro*_1178_-Cas9-*SlTRY* vector **(C)**. Several examples of Sanger sequencinganalysis on the mutation at *SlTRY* targeted sites were given in p*2×35Spro*-Cas9-*SlTRY* and p*AtGCSpro*_1178_-Cas9-*SlTRY* system, respectively **(D)**.

Table S1 All primer sequences used in this paper.

Table S2 Comparison of p*AtGCSpro*_1178_-Cas9-*Rj7*, p*Ubiqutin*_pro_-Cas9-*Rj7*, p*YAO*_pro_-Cas9-*Rj7*, and p*2×35Spro*-Cas9-*Rj7* genome editing efficiency in soybean hairy roots.

Table S3 Comparison of p*AtGCSpro*_1178_-Cas9-*LjNLP4*, p*Ubiqutin*_pro_Cas9-*LjNLP4*, p*YAO*_pro_-Cas9-*LjNLP4*, and p*2×35Spro*-Cas9-*LjNLP4* genome editing efficiency in *L. japonicus* hairy roots.

## Abbreviations

ARM, *Agrobacterium rhizogenes*–mediated transformation; H/BM, Homozygous/Biallelic Mutations; *AtGCS_pro_*, gamma-*glutamylcysteine synthetase* gene promoter; *AtGCSpro*_2411_, *AtGCSpro*_1977_, *AtGCSpro*_1629_, *AtGCSpro*_1178_, *AtGCSpro*_833_, *AtGCSpro* with 2411 bp, 1977 bp, 1629 bp, 1178 bp, and 833 bp, respectively; *2×35Spro*, double CaMV35S promoter; qRT-PCR, quantitative Real-time PCR; PCR/RE, Polymerase chain reaction/restriction enzyme.
