# Supplementary figures and images for: AtGCS promoter-driven clustered regularly interspaced short palindromic repeats/Cas9 highly efficiently generates homozygous/biallelic mutations in the transformed roots by Agrobacterium rhizogenes–mediated transformation
Source: Front Plant Sci. 2022 Oct 18;13:952428. doi: 10.3389/fpls.2022.952428 (PMC9623429; doi:10.3389/fpls.2022.952428)

**FIGURE S1**

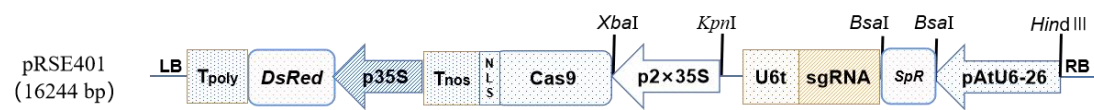

Supplement: Figure S1 — The schematic diagrams of the pRSE401. [file Image_1.pdf]

**FIGURE S2**

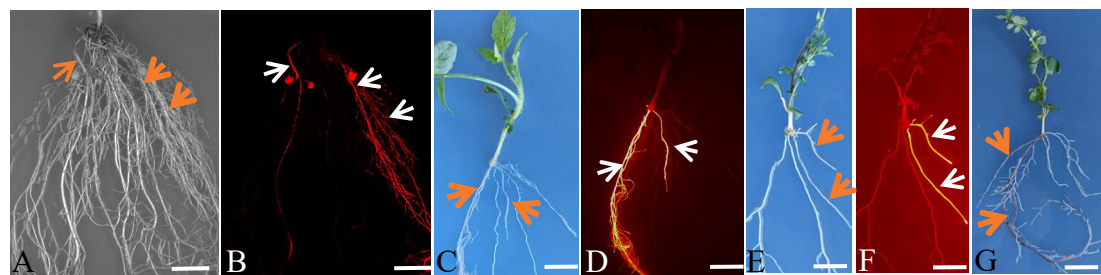

Supplement: Figure S2 — DsRed and AtMyb75 as visual reporter genes used for screening transgenic hairy roots in soybean, tomato, and L. japonicus, respectively. Hairy roots were induced from soybean (A-B), tomato (C-D), and L. japonicas (E-G), respectively. Sections A, C, E, and G were pictured under the bright light. DsRed fluorescence was observed in transgenic positive soybean (B), tomato (D), and L. japonicus (F) hairy roots, and purple/red anthocyannin in L. japonicus (G), respectively. Arrows indicated transgenic positive hairy roots. Bars=5 mm. [file Image_2.pdf]

**FIGURE S3**

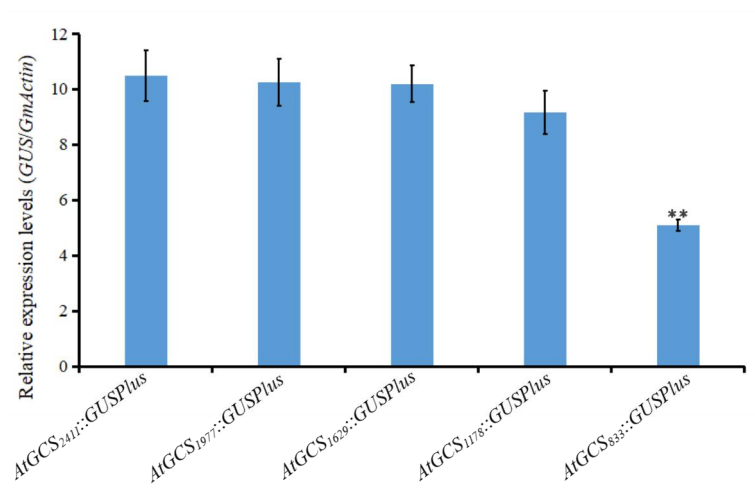

Supplement: Figure S3 — Relative expression levels of GUS in the transformed with pRedGa1/2/3/4 soybean hairy roots by a qRT-PCR assay. Data were analyzed using Microsoft office Excel 2016 and Data Processing System (DPS) statistical software. The averages and standard deviations were calculated. ** represented very significantly different at the p=0.01 value given. [file Image_3.pdf]

FIGURE S5

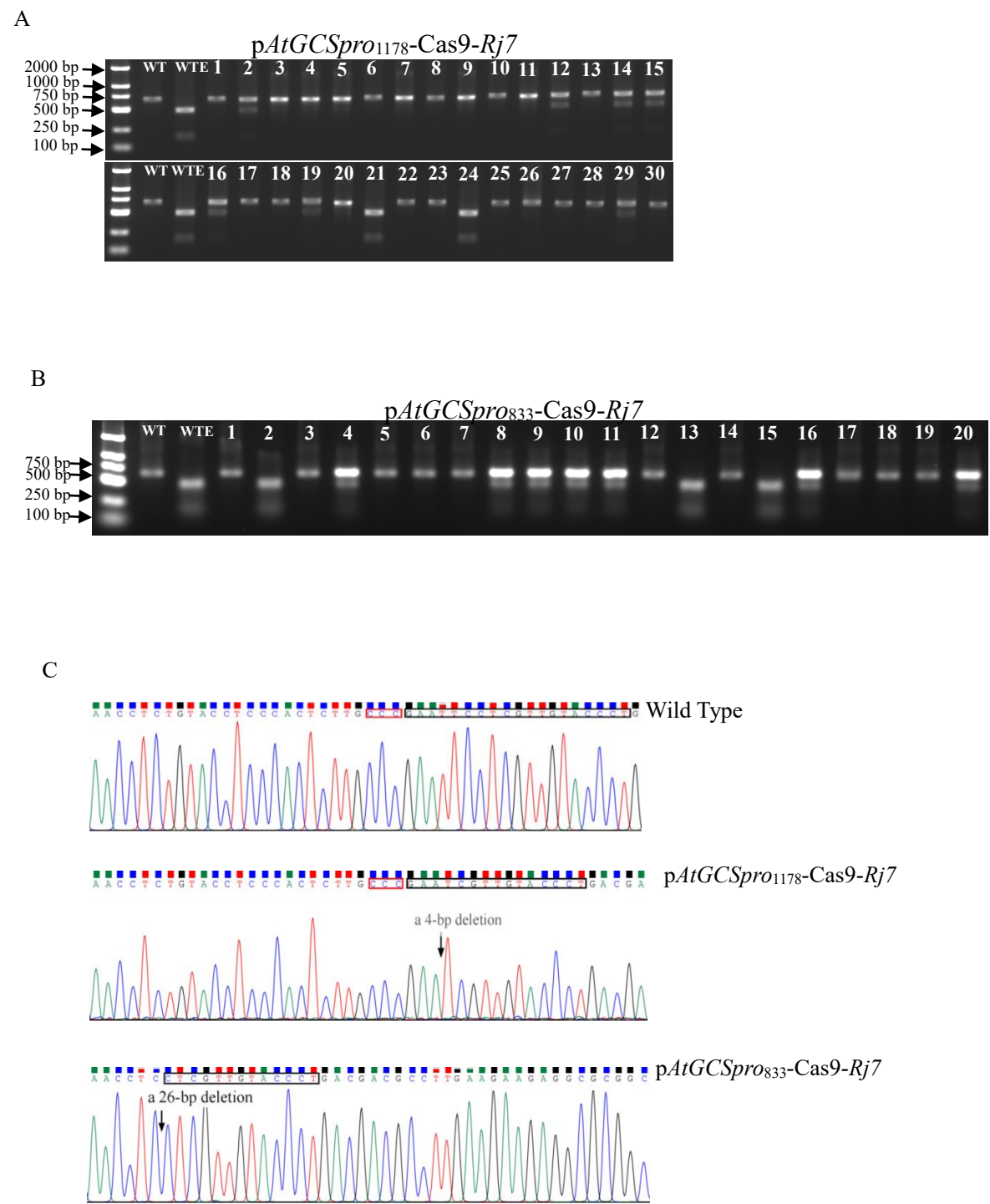

Supplement: Figure S5 — Mutation at Rj7 target site mediated by pAtGCSpro1178-Cas9 and pAtGCSpro833-Cas9 system in soybean, respectively, by PCR-RE assays and Sanger sequencing analysis. PCR-RE assays mutation efficiency in the Rj7 target loci from different independent pAtGCSpro1178-Cas9 and pAtGCSpro833-Cas9 hairy roots, respectively (A, B). Lanes WT and WTE, undigested PCR amplification fragment and digested wild-type controls by EcoRI, respectively. Lanes 1-30, different independent transgenic hairy roots (A). Lane 1, 3-11, 13, 17-18, 20, 22-23, 25-28, and 30 were homozygous/ biallelic mutations (A). Lanes 1-20, different independent transgenic hairy roots (B). Lane 1, 3, 5-7, 12, 14, and 17-19 were homozygous/ biallelic mutations (B). An example of sequencing analysis on the mutation at Rj7 target site was given in pAtGCSpro1178-Cas9 and pAtGCSpro833-Cas9 system, respectively. Black arrows indicate the site of indels mutation. The PAM region and mutated target site are shown in the box (C). [file Image_5.pdf]
