# Supplementary material for: AtGCS promoter-driven clustered regularly interspaced short palindromic repeats/Cas9 highly efficiently generates homozygous/biallelic mutations in the transformed roots by Agrobacterium rhizogenes–mediated transformation
Source: Front Plant Sci. 2022 Oct 18;13:952428. doi: 10.3389/fpls.2022.952428 (PMC9623429; doi:10.3389/fpls.2022.952428)

FIGURE S4

1#

CCCGAA----TCGTTGTACCCTG -4

CCCGAA-------TTGTACCCTG -7

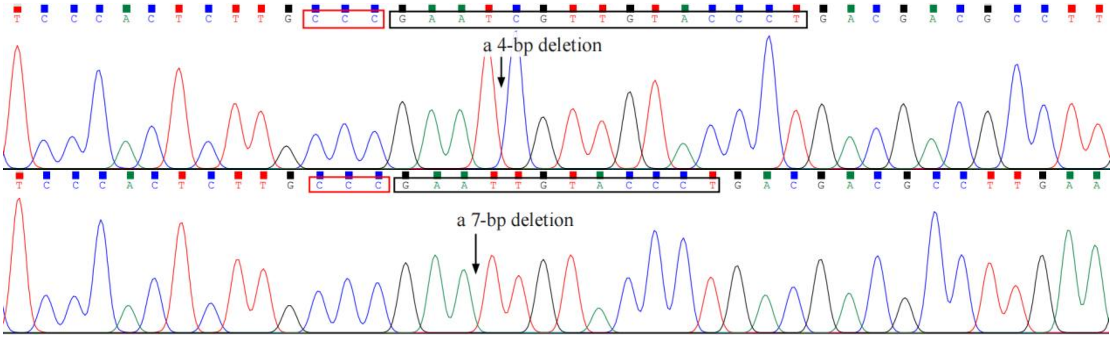

2#

CCCGA---CCTCGTTGTACCCTG -3

CCCGA---CCTCGTTGTACCCTG -3

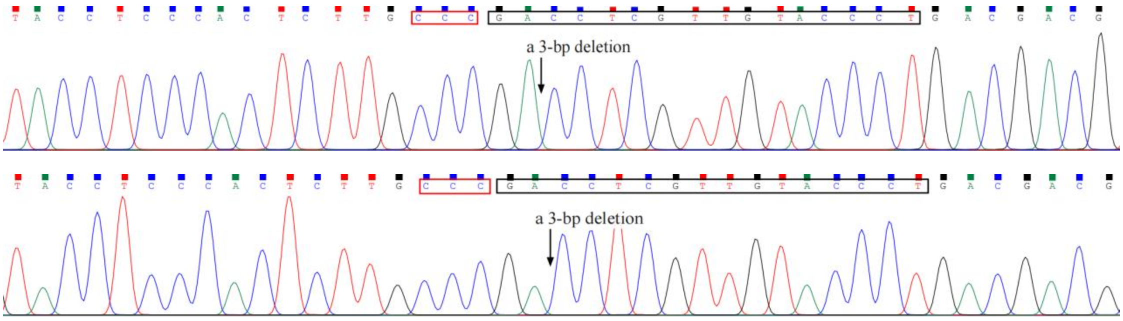

4#

CCCGA-TTCCTCGTTGTACCCTG -1

CCCGA-TTCCTCGTTGTACCCTG -1

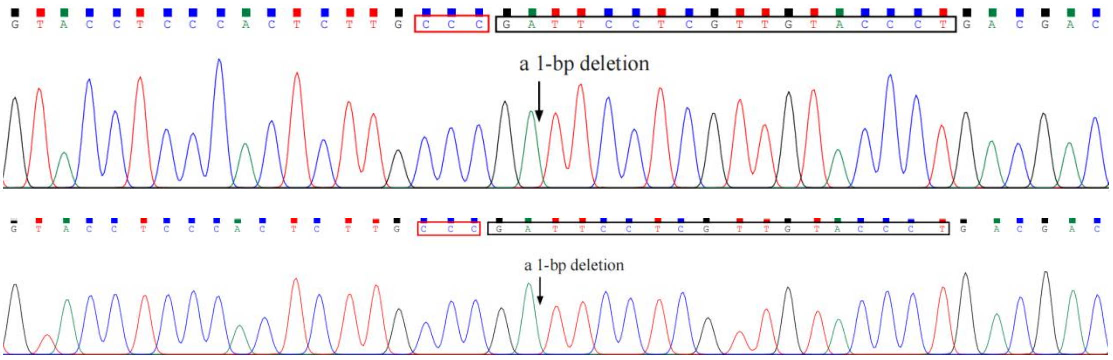

5#

CCCGAA-TCCTCGTTGTACCCTG -1
CCCGA--TCCTCGTTGTACCCTG -2

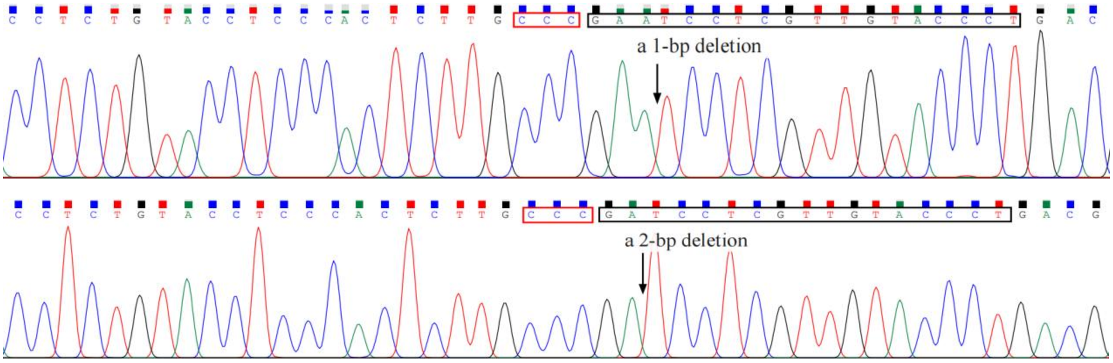

6#

CCCGA----CTCGTTGTACCCTG -4
CCCGA------CGTTGTACCCTG -6

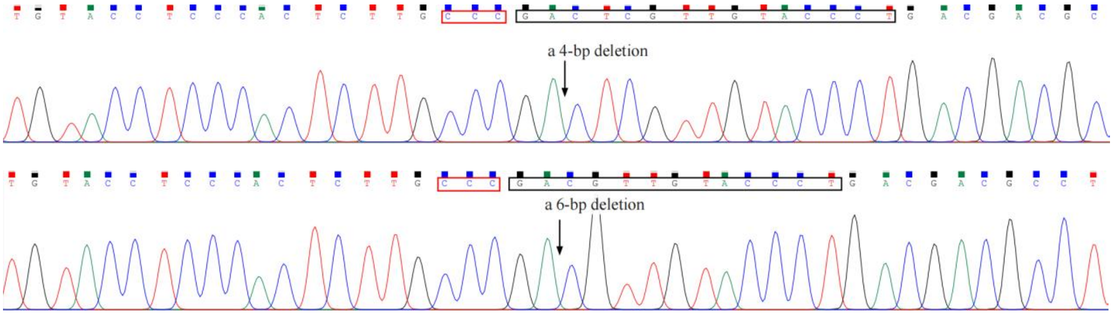

7#

CCCG-----CTCGTTGTACCCTG -5
CCCGAAT--CTCGTTGTACCCTG -2

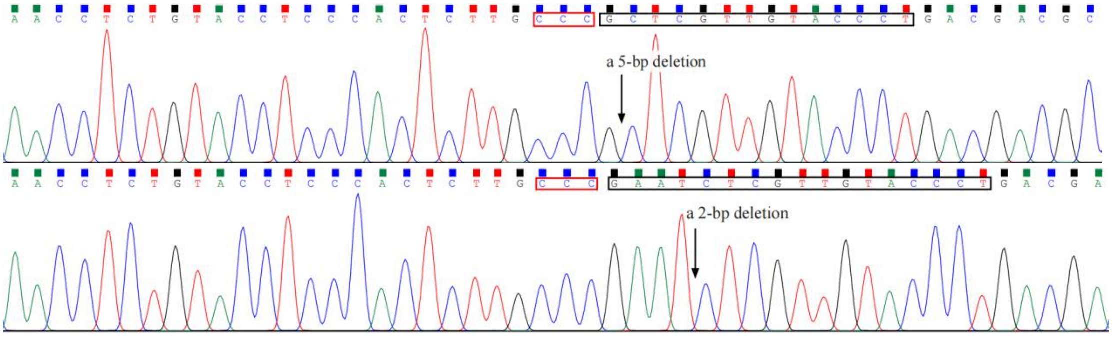

8#

CCCGAAGTTCCTCGTTGTACCCTG +1

CCCGA- --CCTCGTTGTACCCTG -3

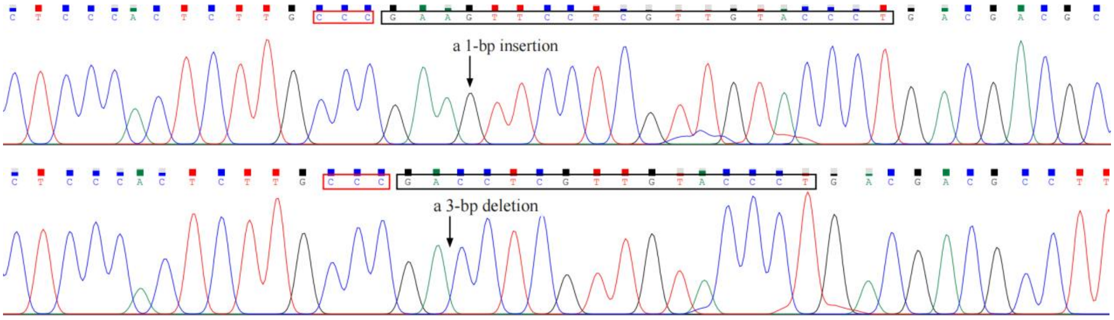

9#

CCCGAAT--CTCGTTGTACCCTG -2

CCCGAA------GTTGTACCCTG -6

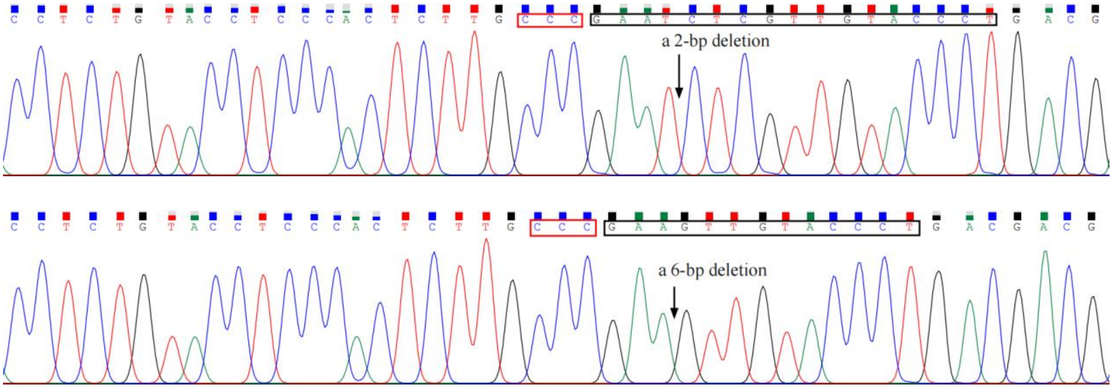

10#

CCCGA-TTCCTCGTTGTACCCTG -1

CCCGA--TCCTCGTTGTACCCTG -2

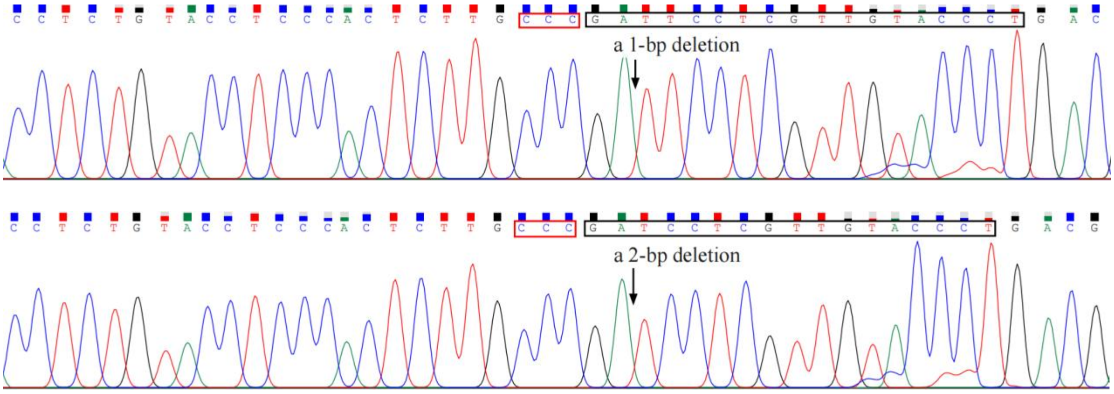

11#

CCCGAA ---CTCGTTGTACCCTG -3

CCCGAGATTCCTCGTTGTACCCTG +1

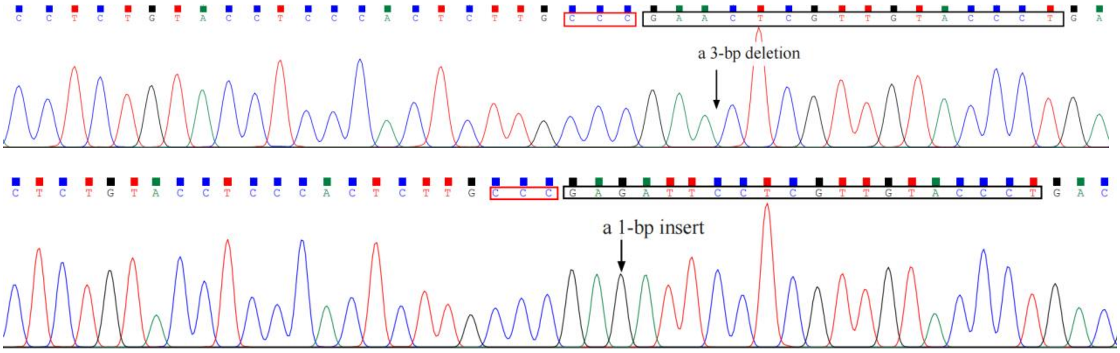

12#

CCCGAAGTTCCTCGTTGTACCCTG +1

CCCGAA ----TCGTTGTACCCTG -4

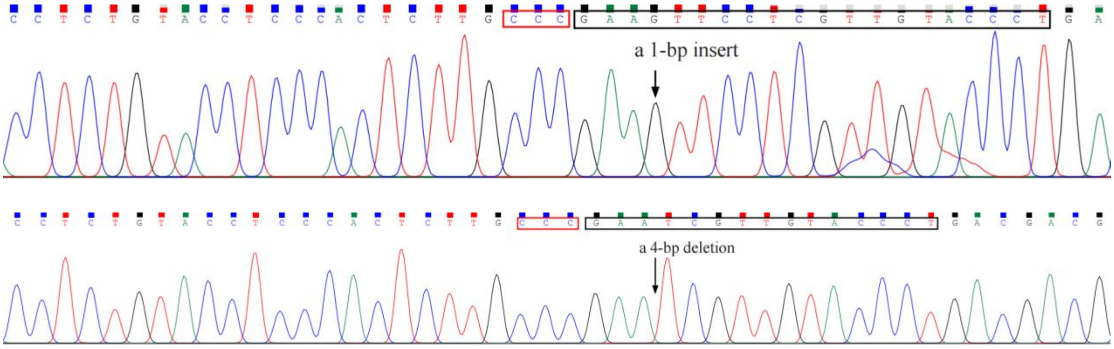

13#

CCCGAGATTCCTCGTTGTACCCTG +1

CCCGAA ---CTCGTTGTACCCTG -3

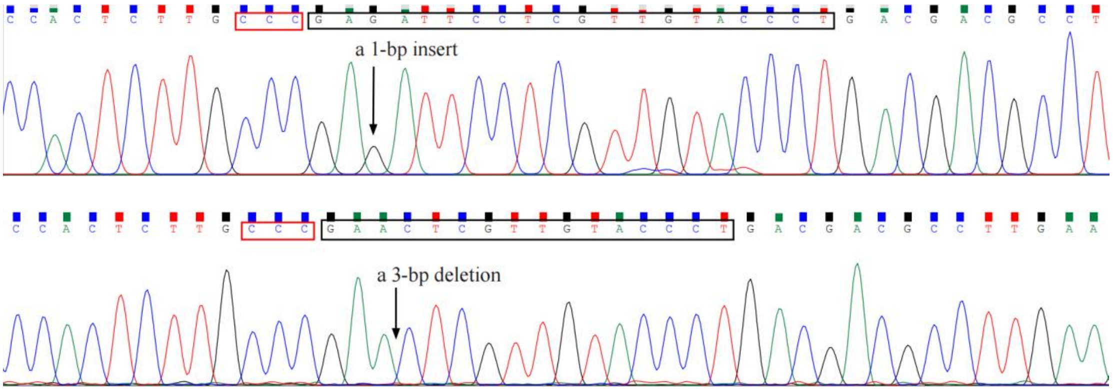

14#

CCCGA---CCTCGTTGTACCCTG -3

CCCGA-TCCCTCGTTGTACCCTG -1

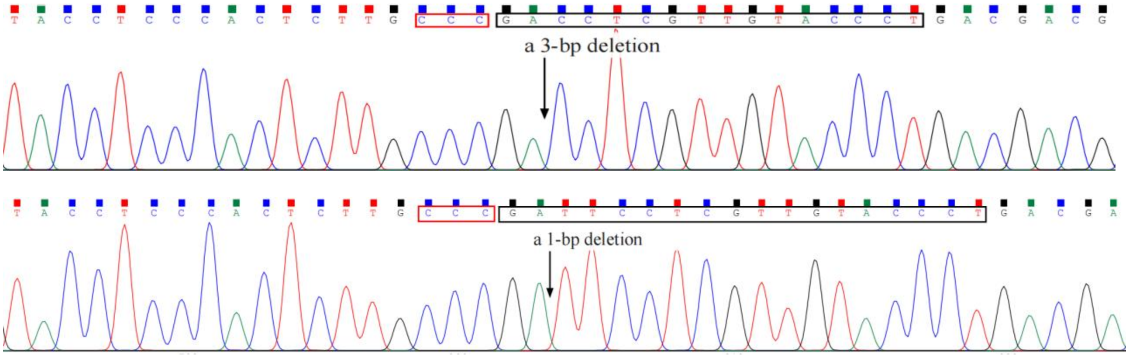

17#

CCCGAA----TCGTTGTACCCTG -4

CCCGA--TCCTCGTTGTACCCTG -2

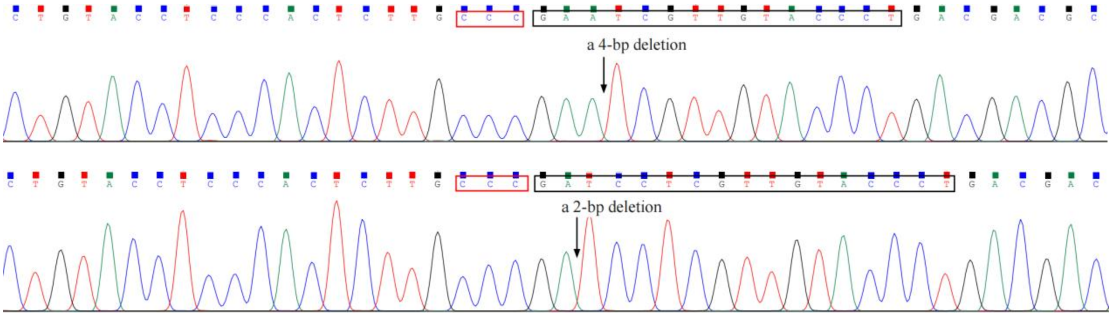

18#

CCCGAA-TCCTCGTTGTACCCTG -1

CCCGAA---CTCGTTGTACCCTG -3

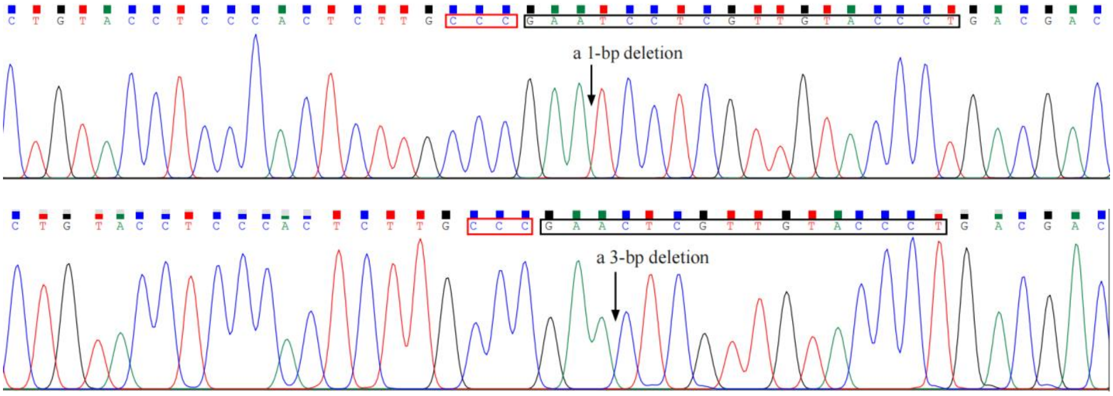

19#

CCCGA-------GTTGTACCCTG -7
CCCGA--TCCTCGTTGTACCCTG -2

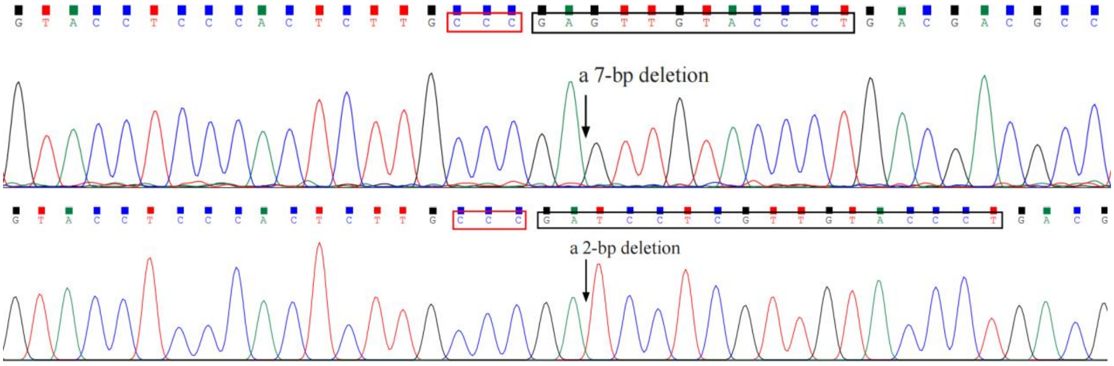

21#

CCCGA--TCCTCGTTGTACCCTG -2
CCCGA---CCTCGTTGTACCCTG -3

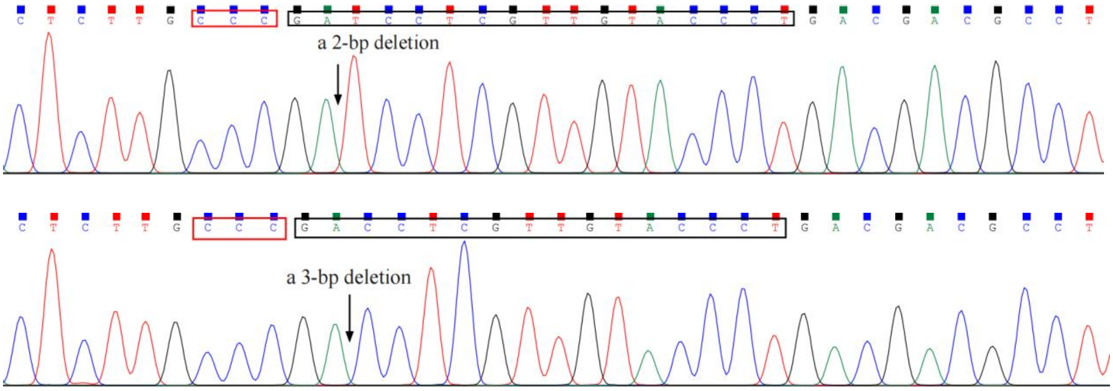

22#

CCCGAA------GTTGTACCCTG -6
CCCGA--------TTGTACCCTG -8

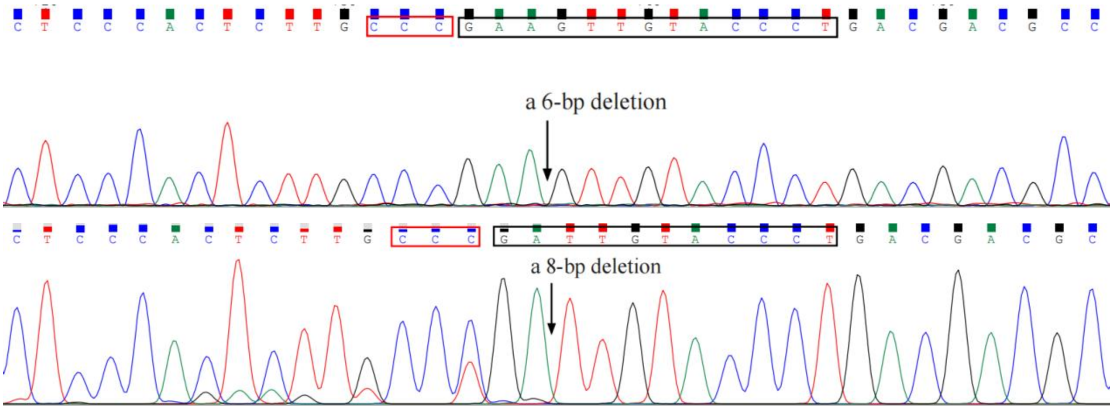

24#

CCCGAGATTCCTCGTTGTACCCTG +1

CCCGAA------GTTGTACCCTG -6

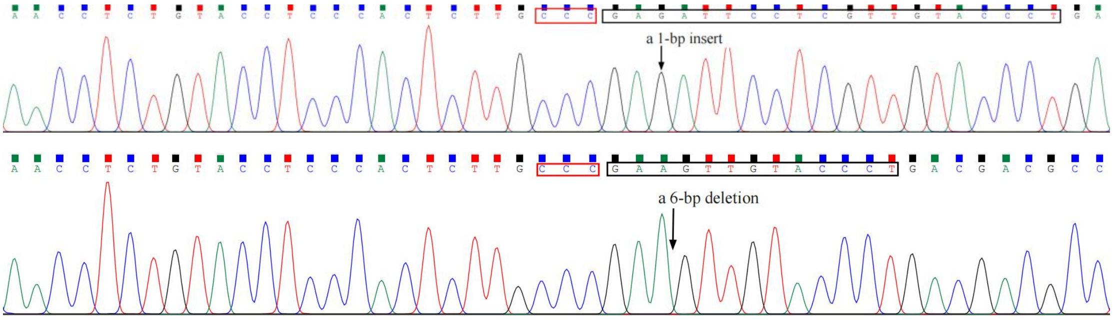

25#

CCCGA-----TCGTTGTACCCTG -5

CCCGA----CTCGTTGTACCCTG -4

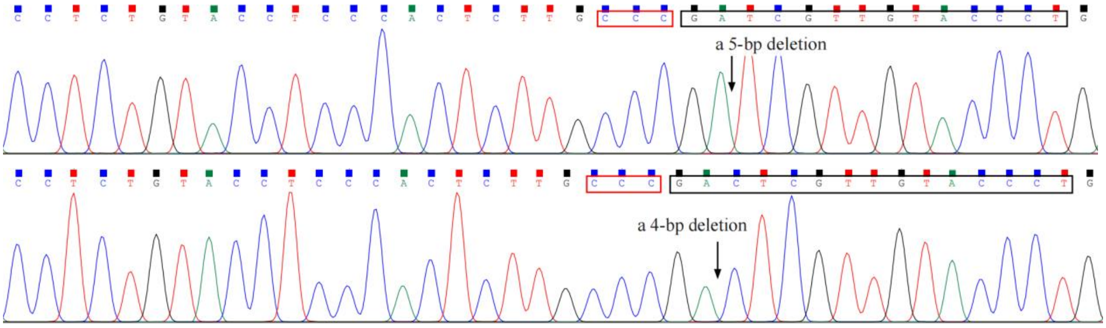

26#

CCCGAA-TCCTCGTTGTACCCTG -1

CCCG-----CTCGTTGTACCCTG -5

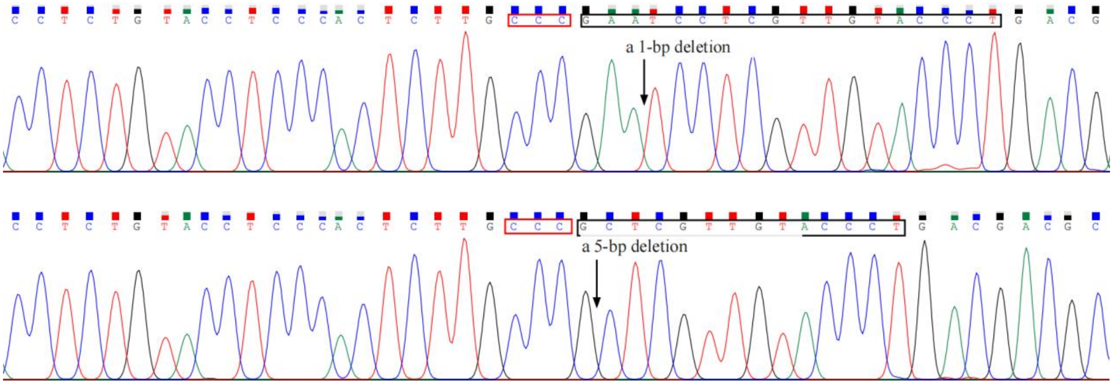

29#

CCCG-----CTCGTTGTACCCTG -5

CCCGA-------GTTGTACCCTG -7

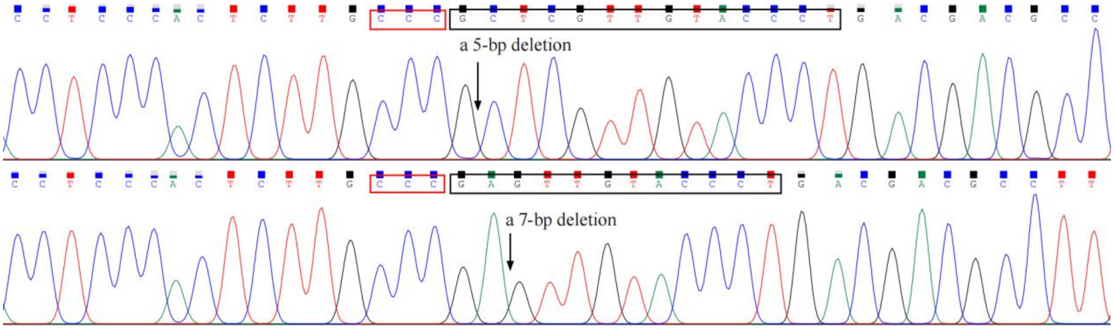

30#

CCCGAA---CTCGTTGTACCCTG -3

CCCGAA------GTTGTACCCTG -6

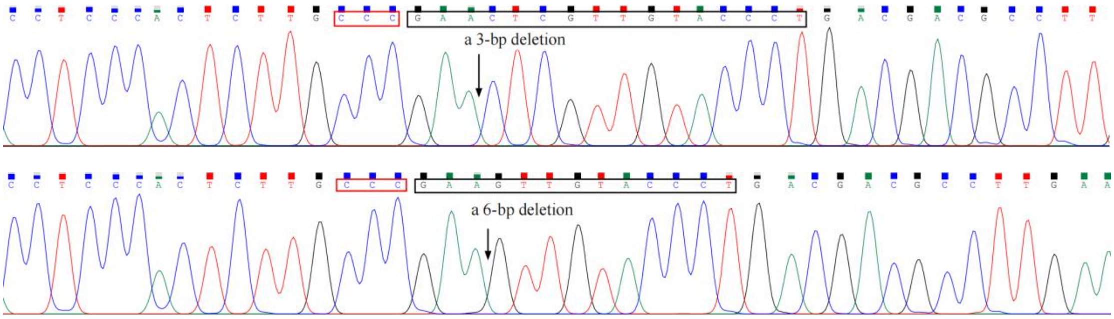

Supplement: Figure S4 — Sanger sequencing chromatogram of analysis on the H/BM mutations at 15 Rj7 target site mediated by pAtGCSpro2411-Cas9-Rj7 system were given. [file Image_4.pdf]
