# Supplementary material for: AtGCS promoter-driven clustered regularly interspaced short palindromic repeats/Cas9 highly efficiently generates homozygous/biallelic mutations in the transformed roots by Agrobacterium rhizogenes–mediated transformation
Source: Front Plant Sci. 2022 Oct 18;13:952428. doi: 10.3389/fpls.2022.952428 (PMC9623429; doi:10.3389/fpls.2022.952428)
Supplement: Figure S6 — Mutation at GmNNL1Rfg1 target sites in soybean mediated by p2×35Spro-Cas9 and pAtGCSpro1178-Cas9 system, respectively, and PCR-RE assays and Sanger sequencing analysis. Sequence of an sgRNA designed to target a site within the fifth exon region of GmNNL1 (A) and Rfg1 (B), respectively. The HindIII (A) and NcoI (B) restriction site is underlined, respectively. The PAM sequence is highlighted in blue. PCR-RE analysis the targeted mutation at GmNNL1 site using HindIII restriction enzyme digestion. In the hairy roots transformed with the p2×35Spro-Cas9-GmNNL1Rfg1 vector, no homozygous or biallelic mutations lines were obtained among 30 independent transgenic lines (C). PCR-RE analysis the targeted mutation at Rfg1 site using NcoI restriction digestion. In the hairy roots transformed with the p2×35Spro-Cas9-GmNNL1Rfg1 vector, 41 10 lines (#5-7, #11, #14-17, #19, and #26) were homozygous or biallelic mutations among 30 independent transgenic lines (D). PCR-RE analysis the targeted mutation in GmNNL1 site in the hairy roots transformed with the pAtGCSpro1178-Cas9-GmNNL1Rfg1 vector using HindIII restriction enzyme digestion. 2 lines (#28, and #30) were homozygous or biallelic mutations among independent transgenic lines (E). PCR-RE analysis the targeted mutation at Rfg1 site in the hairy roots transformed with the pAtGCSpro1178-Cas9-GmNNL1Rfg1 vector using NcoI restriction enzyme digestion. 25 lines (#1-4, #7-11, #13-15, #17-28, and #30 ) were homozygous or biallelic mutations among 30 independent transgenic lines (F). An example of sequencing analysis on the mutation at GmNNL1 target site was given in pAtGCSpro1178-Cas9-GmNNL1Rfg1 and p2×35Spro-Cas9-GmNNL1Rfg1 system, respectively. WT represents wild type. Black arrows indicate the site of indels mutation (G). An example of sequencing analysis on the mutation at Rfg1 target site was given in pAtGCSpro1178-Cas9-GmNNL1Rfg1 and p2×35Spro-Cas9-GmNNL1Rfg1 system, respectively. Black arrows indicate the site of indels mutation ( [file Image_6.pdf]

FIGURE S6

A

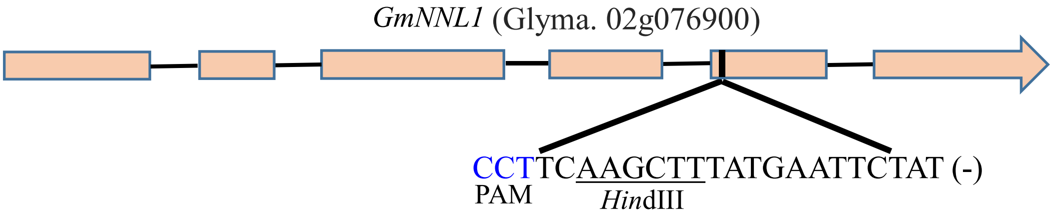

B

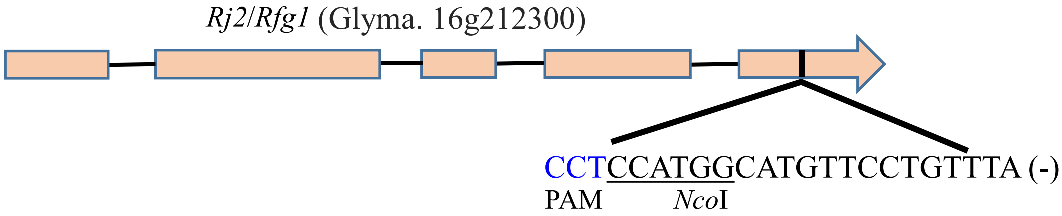

C

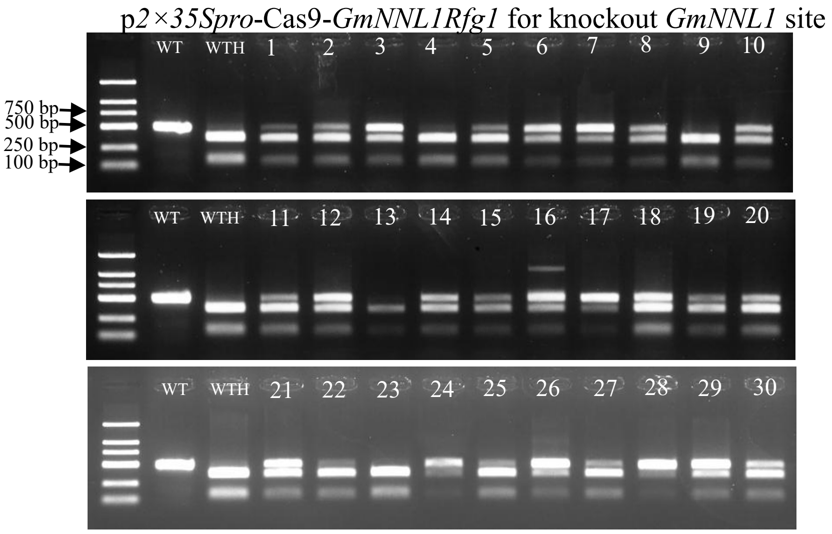

D

p*2×35Spro*-Cas9-*GmNNL1Rfg1* for knockout *Rfg1* site

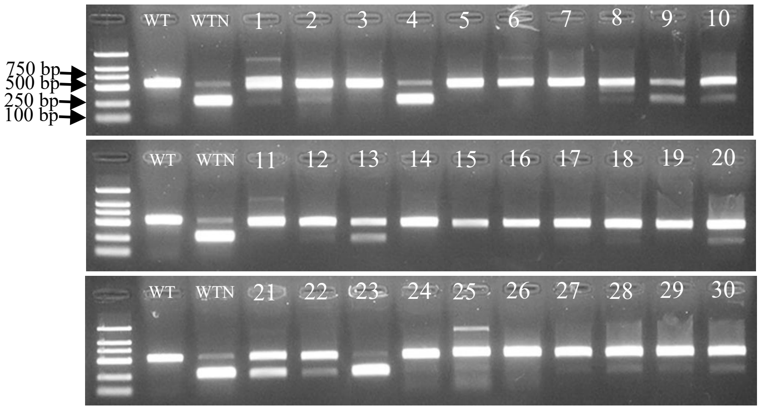

E

p*AtGCSpro*$_{1178}$-Cas9-*GmNNL1Rfg1* for knockout *GmNNL1* site

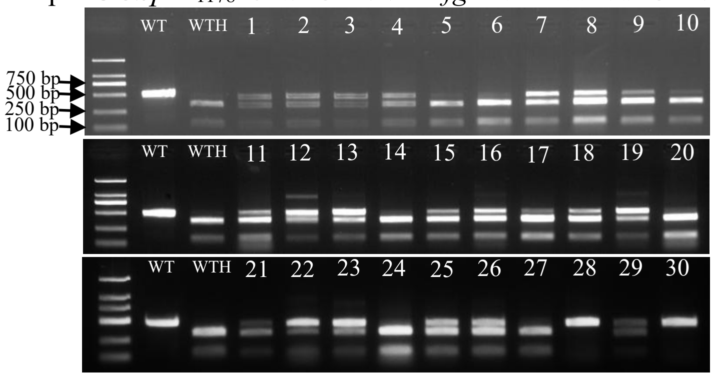

F

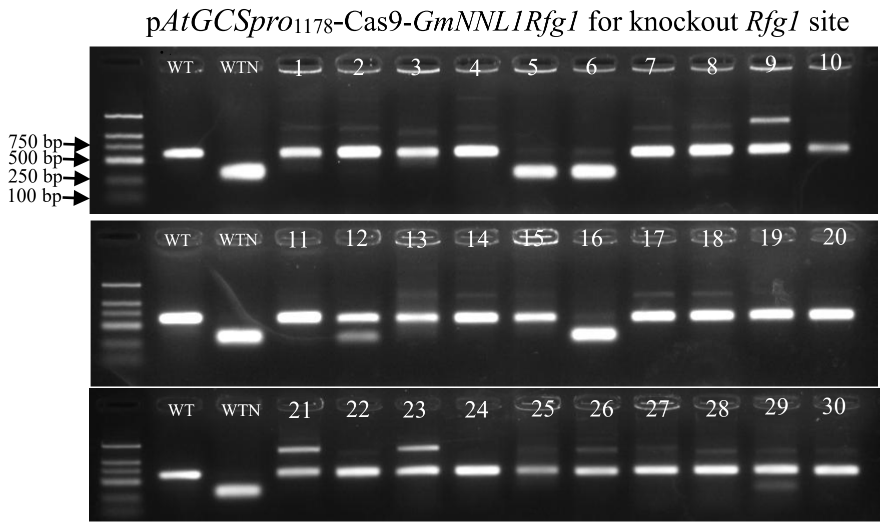

pAtGCSpro1178-Cas9-GmNNL1Rfg1 for knockout Rfg1 site
WT
WTN
1 2 3 4 5 6 7 8 9 10
11 12 13 14 15 16 17 18 19 20
21 22 23 24 25 26 27 28 29 30
750 bp
500 bp
250 bp
100 bp

G

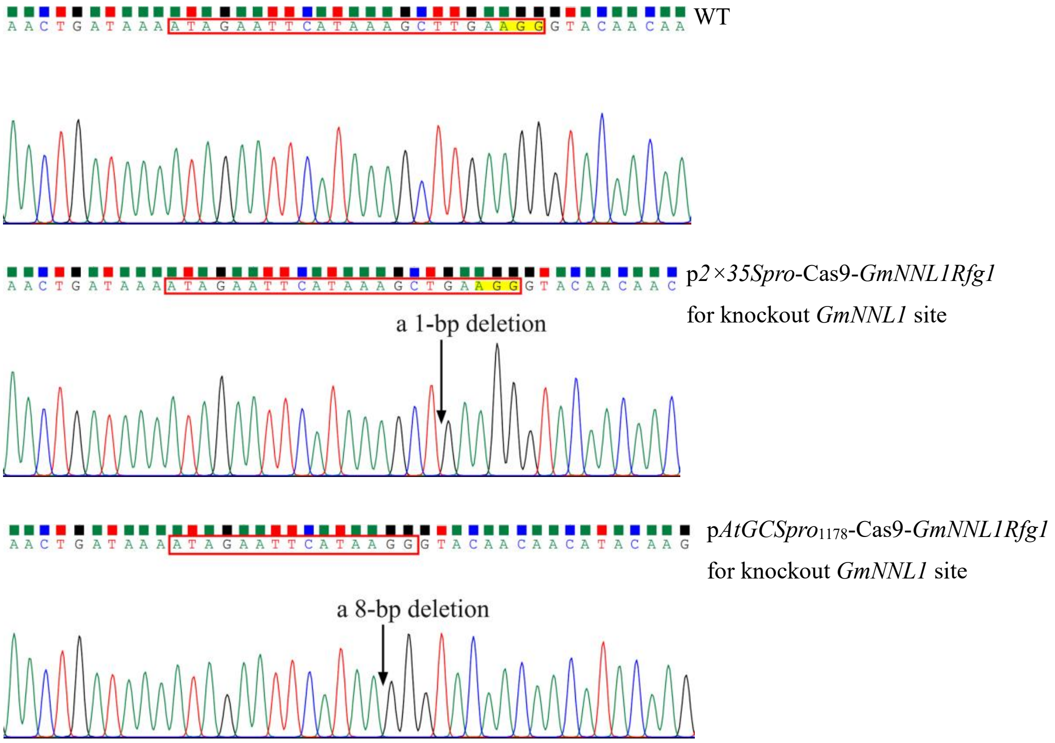

AACTGATAAAATAGAATTCATAAAGCTTGAAGGGTACAACAA
WT
AACTGATAAAATAGAATTCATAAAGCTGAAGGGTACAACAAC
p2×35Spro-Cas9-GmNNL1Rfg1
for knockout GmNNL1 site
a 1-bp deletion
AACTGATAAAATAGAATTCATAAGGGTACAACAACATACAAG
pAtGCSpro1178-Cas9-GmNNL1Rfg1
for knockout GmNNL1 site
a 8-bp deletion

H

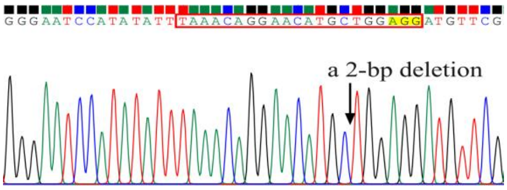

p*2×35Spro*-Cas9-*GmNNL1Rfg1*
for knockout *Rfg1* site

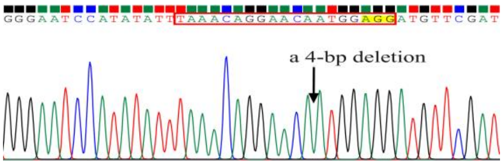

p*AtGCSpro*$_{1178}$-Cas9-*GmNNL1Rfg1*
for knockout *Rfg1* site
