# Supplementary material for: AtGCS promoter-driven clustered regularly interspaced short palindromic repeats/Cas9 highly efficiently generates homozygous/biallelic mutations in the transformed roots by Agrobacterium rhizogenes–mediated transformation
Source: Front Plant Sci. 2022 Oct 18;13:952428. doi: 10.3389/fpls.2022.952428 (PMC9623429; doi:10.3389/fpls.2022.952428)
Supplement: Figure S7 — Mutation at LjNLP4LjSYMRK target sites in L. japonicus mediated by p2×35Spro-Cas9 and pAtGCSpro1178-Cas9 system, respectively, and PCR-RE assays and Sanger sequencing analysis. Sequence of an sgRNA designed to target a site within the first exon region of LjNLP4 (A) and within the thirteenth exon region of LjSYMRK (B), respectively. The BamHI (A) and PstI (B) restriction site is underlined, respectively. The protospaceradjacent motif (PAM) sequence is highlighted in blue. PCR-RE assays to detect CRISPR/Cas9-induced mutation in the LjNLP4 target loci using BamHI from 30 different independent p2×35Spro-Cas9 LjNLP4LjSYMRK (C) and pAtGCSpro1178-Cas9-LjNLP4LjSYMRK (D) hairy roots, respectively. 9 lines (#3, #4, #9, #10, #12, #17, #21, #24, and #29) were homozygous or biallelic mutations (C). 25 lines (#2-7, #9-15, #17-24, #26-28, and #30) were homozygous or biallelic mutations (D). PCR-RE assays to detect CRISPR/Cas9-induced mutation in the LjSYMRK target loci using PstI from 30 different independent p2×35Spro-Cas9-LjNLP4LjSYMRK (E) and pAtGCSpro1178-Cas9-LjNLP4LjSYMRK (F) hairy roots, respectively. 8 lines (#1, #4, #8, #12, #19, #22, #24, and #28) were homozygous or biallelic mutations (E). 25 lines (#1-6, #8-14, #16, #18-20, and #22-29) were homozygous or biallelic mutations (F). Several examples of sequencing analysis on the mutation at LjSYMRK target site was given in p2×35Spro-Cas9 and pAtGCSpro1178-Cas9 system, respectively (G). [file Image_7.pdf]

FIGURE S7

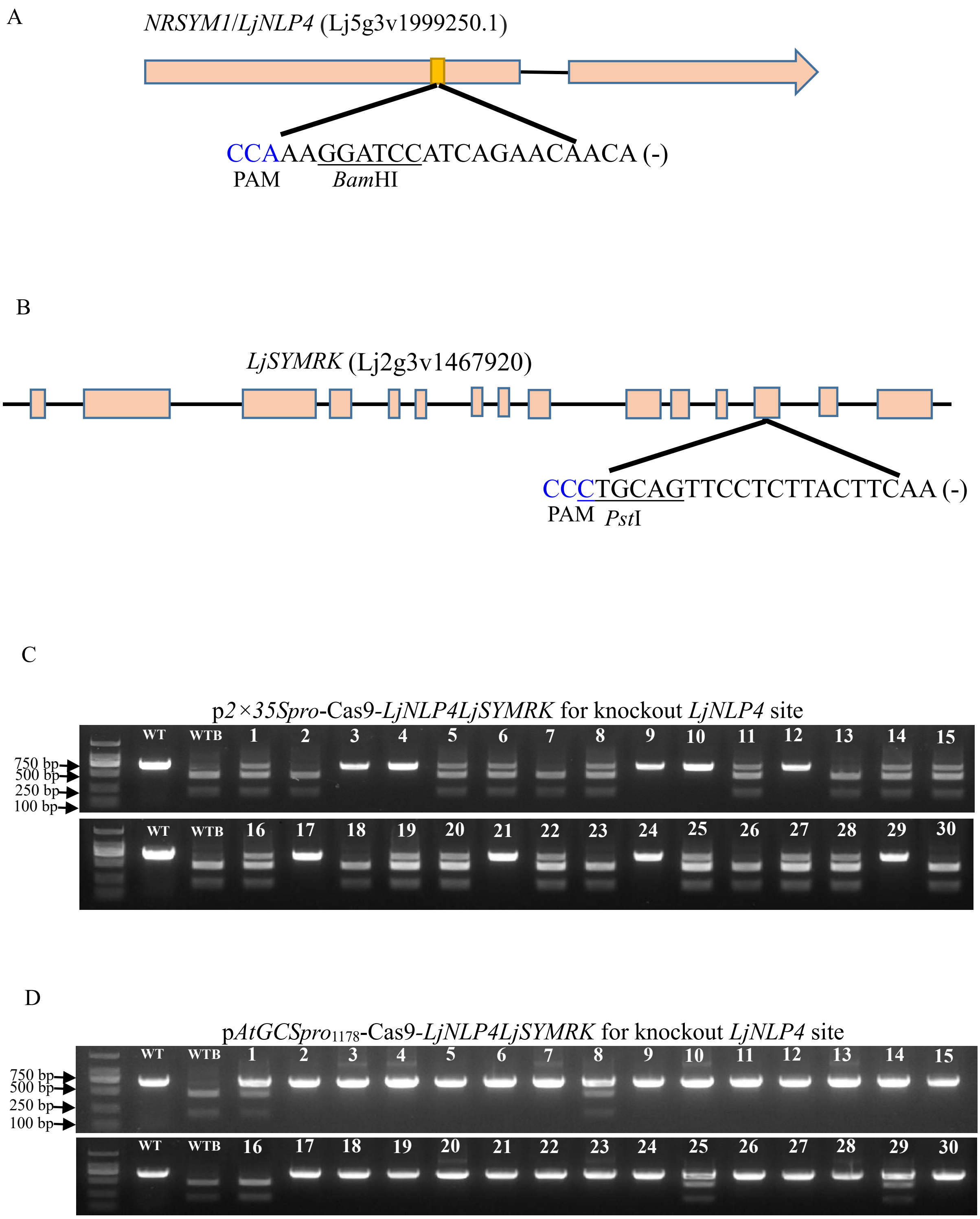
A
NRSYM1/LjNLP4 (Lj5g3v1999250.1)
CCAAAGGATCCATCAGAACAACA (-)
PAM
BamHI
B
LjSYMRK (Lj2g3v1467920)
CCCTGCAGTTCCTCTTACTTCAA (-)
PAM
PstI
C
p2×35Spro-Cas9-LjNLP4LjSYMRK for knockout LjNLP4 site
750 bp
500 bp
250 bp
100 bp
WT WTB 1 2 3 4 5 6 7 8 9 10 11 12 13 14 15
WT WTB 16 17 18 19 20 21 22 23 24 25 26 27 28 29 30
D
pAtGCSpro1178-Cas9-LjNLP4LjSYMRK for knockout LjNLP4 site
750 bp
500 bp
250 bp
100 bp
WT WTB 1 2 3 4 5 6 7 8 9 10 11 12 13 14 15
WT WTB 16 17 18 19 20 21 22 23 24 25 26 27 28 29 30

E

p*2×35Spro*-Cas9-*LjNLP4LjSYMRK* for knockout *LjSYMRK* site

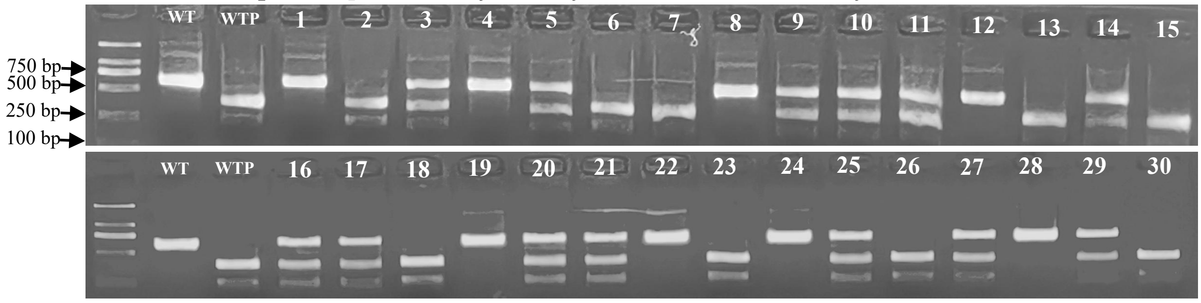

F

p*AtGCSpro*$_{1178}$-Cas9-*LjNLP4LjSYMRK* for knockout *LjSYMRK* site

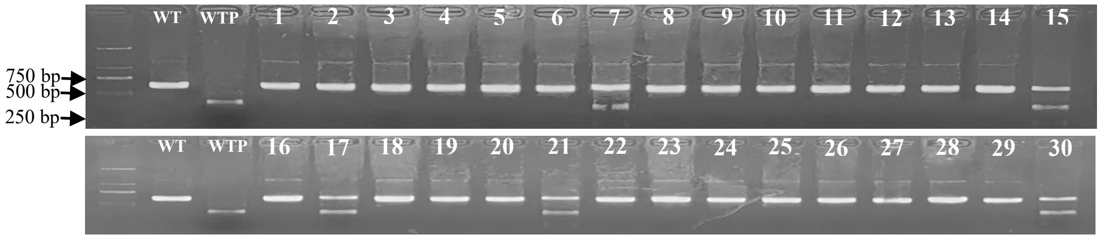

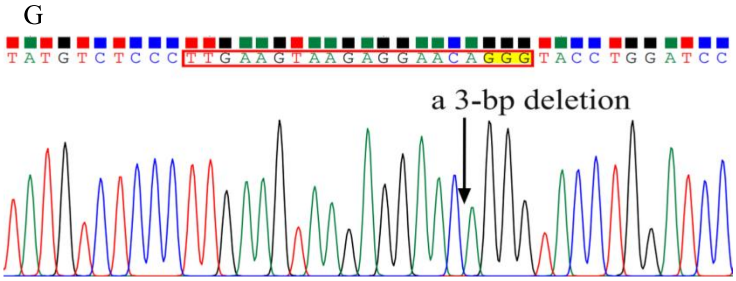

p*2×35Spro*-Cas9-*LjNLP4LjSYMRK*
for knockout *LjSYMRK* site

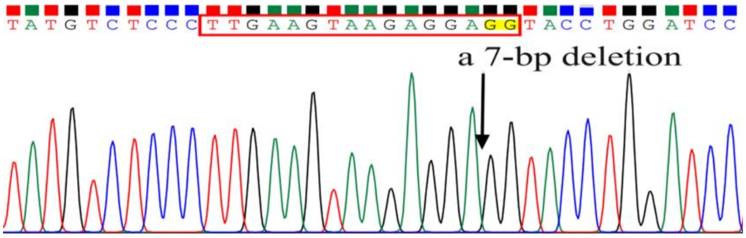

p*2×35Spro*-Cas9-*LjNLP4LjSYMRK*
for knockout *LjSYMRK* site

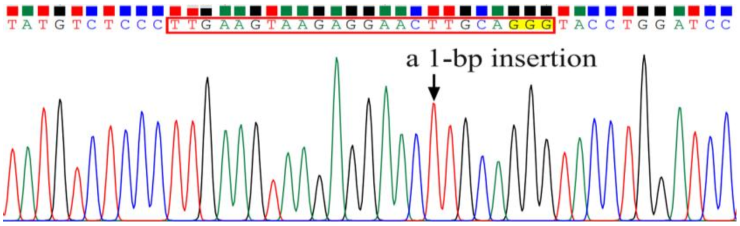

p*AtGCSpro*$_{1178}$-Cas9-*LjNLP4LjSYMRK*
for knockout *LjSYMRK* site

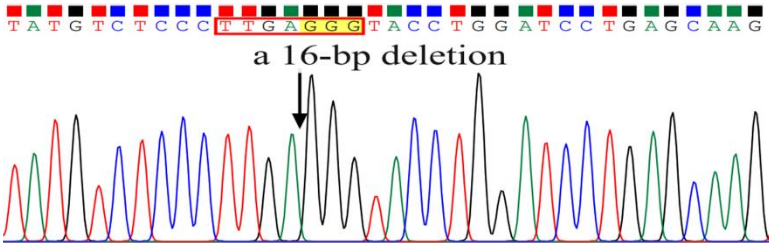

p*AtGCSpro*$_{1178}$-Cas9-*LjNLP4LjSYMRK*
for knockout *LjSYMRK* site
