# Supplementary material for: AtGCS promoter-driven clustered regularly interspaced short palindromic repeats/Cas9 highly efficiently generates homozygous/biallelic mutations in the transformed roots by Agrobacterium rhizogenes–mediated transformation
Source: Front Plant Sci. 2022 Oct 18;13:952428. doi: 10.3389/fpls.2022.952428 (PMC9623429; doi:10.3389/fpls.2022.952428)

FIGURE S8

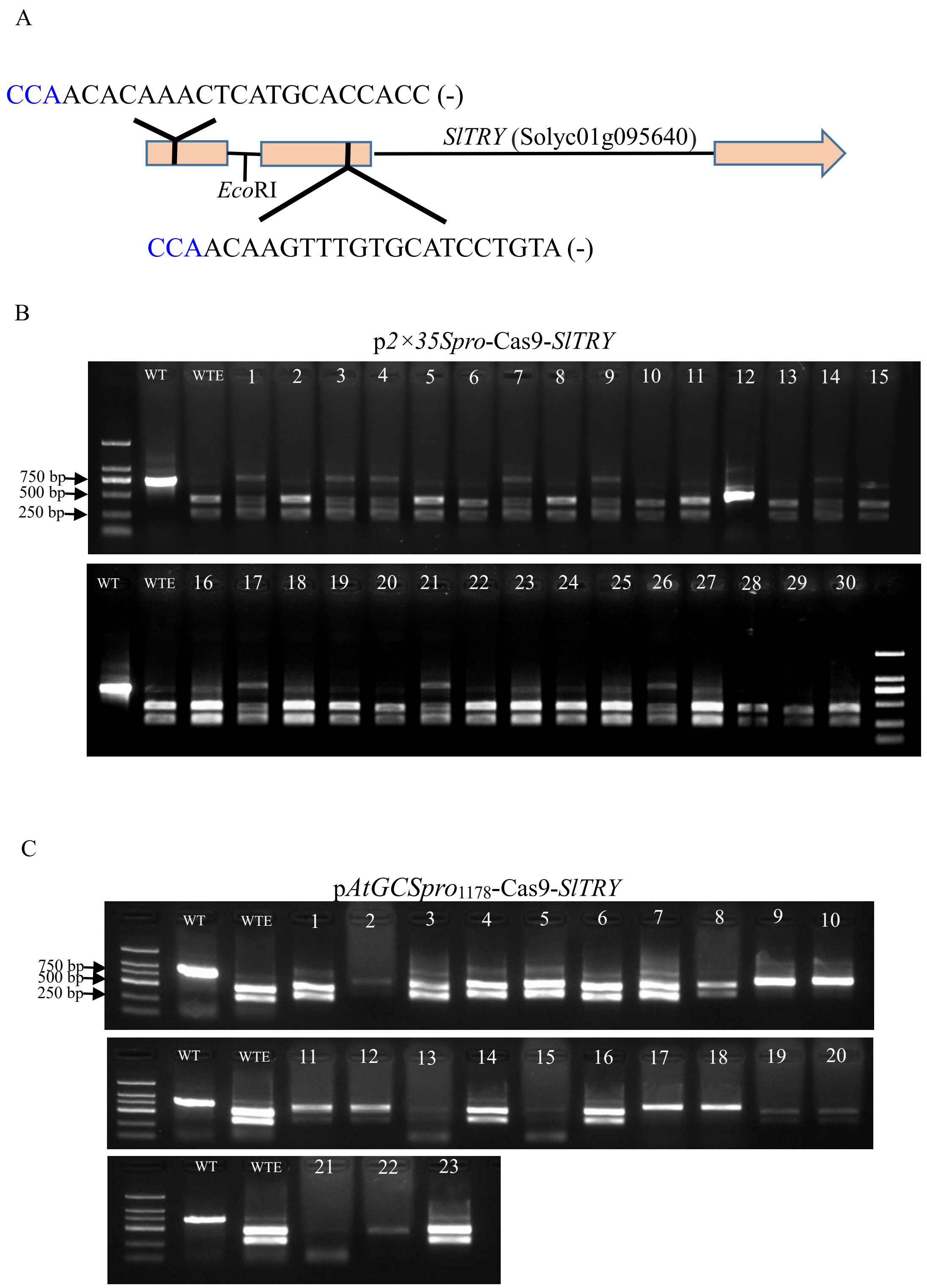
A
CCAACACAAACTCATGCACCACC (-)
SlTRY (Solyc01g095640)
EcoRI
CCAACAAGTTTGTGCATCCTGTA (-)
B
p2×35Spro-Cas9-SlTRY
WT WTE 1 2 3 4 5 6 7 8 9 10 11 12 13 14 15
750 bp
500 bp
250 bp
WT WTE 16 17 18 19 20 21 22 23 24 25 26 27 28 29 30
C
pAtGCSpro1178-Cas9-SlTRY
WT WTE 1 2 3 4 5 6 7 8 9 10
750 bp
500 bp
250 bp
WT WTE 11 12 13 14 15 16 17 18 19 20
WT WTE 21 22 23

D

Line #T1 target site 1: a 1 bp insertion; target site 2: a 73 bp deletion)

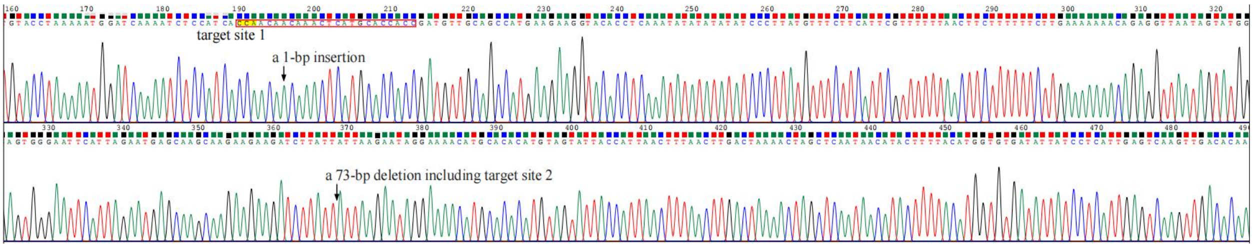

line#T3-2 (target site 1: a 1 bp insertion; target site 2: a 7 bp deletion)

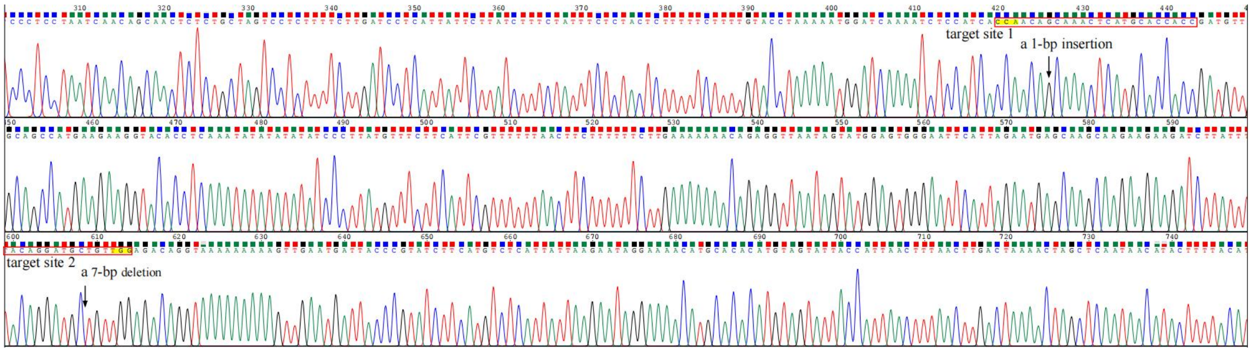

line#T3-3 (target site 1: a 1 bp deletion; target site 2: a 175 bp deletion and a 11 bp insertion)

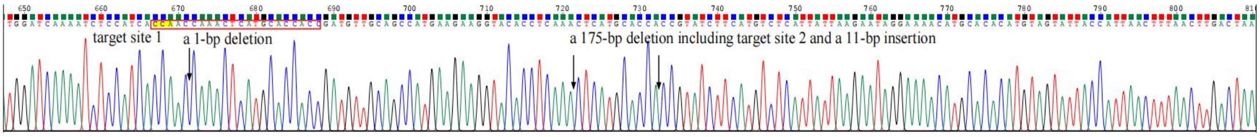

line#T9 (target site 1 and 2: a 189 bp deletion)

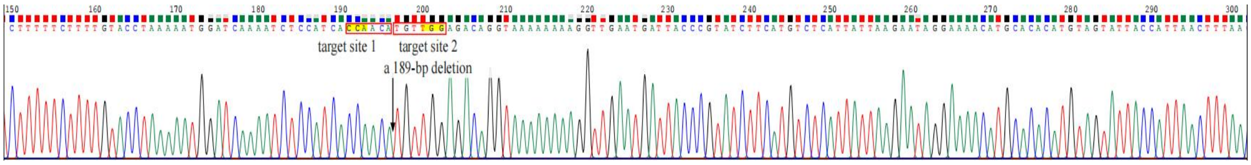

line#T13-3 (target site 1: a 3 bp deletion; target site 2: a 1 bp deletion)

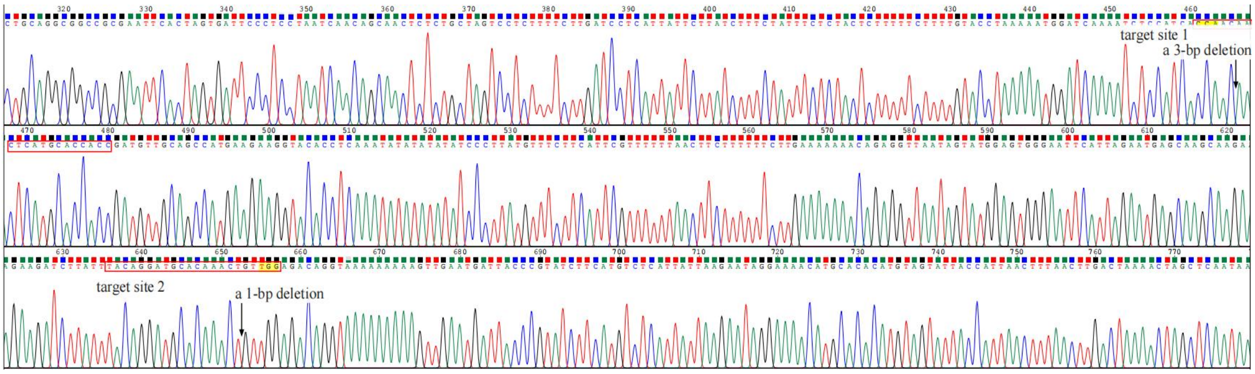

line#T16-5 (target site 1: a 1 bp insertion; target site 2: a 74 bp deletion)

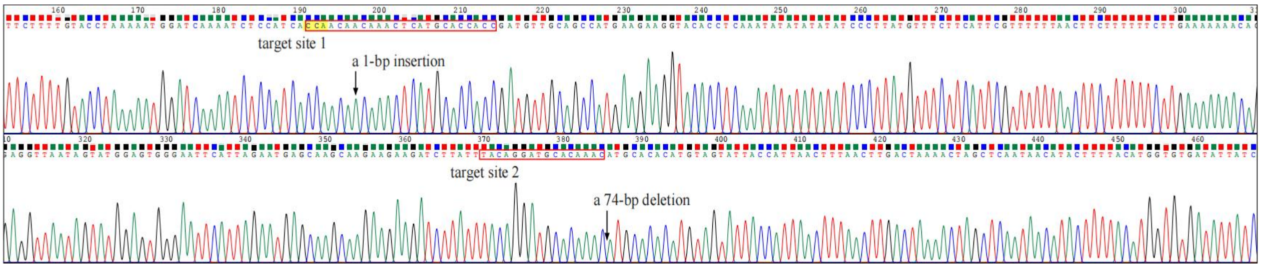

line#T18-2 (target site 1: a 1 bp insertion; target site 2: a 107 bp deletion)

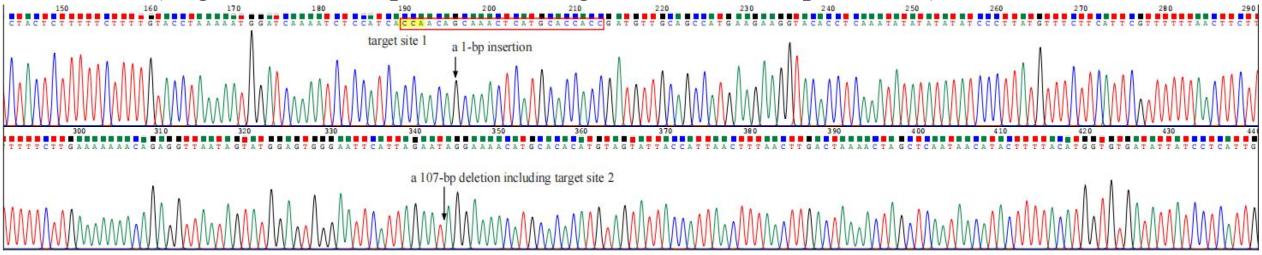

Supplement: Figure S8 — Mutation at SlTRY target sites in tomato mediated by p2×35Spro-Cas9 and pAtGCSpro1178-Cas9 system, respectively, and PCR-RE assays and Sanger sequencing analysis. Sequences of two sgRNA designed to target sites within the first and second exons region of SlTRY. The PAM sequence is highlighted in blue and the EcoRI restriction site is underlined (A). PCR-RE analysis the targeted mutation at SlTRY site using EcoRI restriction enzyme digestion. In the hairy roots transformed with the p2×35Spro-Cas9-SlTRY vector (B). PCR-RE analysis the targeted mutation at SlTRY site using EcoRI restriction digestion. In the hairy roots transformedwith the pAtGCSpro1178-Cas9-SlTRY vector (C). Several examples of Sanger sequencinganalysis on the mutation at SlTRY targeted sites were given in p2×35Spro-Cas9-SlTRY and pAtGCSpro1178-Cas9-SlTRY system, respectively (D). [file Image_8.pdf]
