# Supplementary material for: AtGCS promoter-driven clustered regularly interspaced short palindromic repeats/Cas9 highly efficiently generates homozygous/biallelic mutations in the transformed roots by Agrobacterium rhizogenes–mediated transformation
Source: Front Plant Sci. 2022 Oct 18;13:952428. doi: 10.3389/fpls.2022.952428 (PMC9623429; doi:10.3389/fpls.2022.952428)
Supplement: Table S1 — All primer sequences used in this paper. [file Table_1.pdf]

Table S1. All primer sequences used in this paper.

primers name	Sequence (5'-3')	purposes
GaBa1	ctgcGGATCCATGTGTAGTTGGGCTTCGGT	forward primer for cloning 2430 bp *AtGCSpro* promoter
GaBa2	ctgcGGATCCAGGAAGAAACTGTGGAGGTG	forward primer for cloning 1990 bp *AtGCSpro* promoter
GaBa3	ctgcGGATCCACAGCTTCATAATTCATAGGAG	forward primer for cloning 1640 bp *AtGCSpro* promoter
GaBa4	ctgcGGATCCACCATGGTACCATACTATTCC	forward primer for cloning 1200 bp *AtGCSpro* promoter
GaBa5	ctgcGGATCCTGAGGTCATGTGGTTGGTTAG	forward primer for cloning 850 bp *AtGCSpro* promoter
GaBNR	gttcGGTCTCCCATGGTATATATAGCTCCTGCAATT	reverse primer for cloning *AtGCSpro* promoter
Sap401	CCGCTCTTCAGCTAGGATCCATGGAATCGGCAG CAAAGGA	construction of intermediate vector pRd35SCas9
Rd4012	TGGCTCGAAGATACCTGCAAGAGGTCTCCTGA CCAATGGTGCTTTGTAG	construction of intermediate vector pRd35SCas9
Rd4013	GACCTCTTGCAGGTATCTTCGAGCCA	construction of intermediate vector pRd35SCas9
Sap402	ATGCTCTTCtGCTGGTACCGCTTATTGGTTTATCT CATCGG	construction of intermediate vector pRd35SCas9
GaK1	ACGGTCTCAGTACATGTGTAGTTGGGCTTCGGT	construction of intermediate vector pRdGa1Cas9
GaBa4	CTGCGGATCCACCATGGTACCATACTATTCC	construction of intermediate vector pRdGa4Cas9
GaK5	GTTAGGTACCTGAGGTCATGTGGTTGGTTAG	construction of intermediate vector pRdGa5Cas9
GaX2	GCGTCTAGAGGTATATATAGCTCCTGCAATT	construction of intermediate vector pRdGa1Cas9, pRdGa4Cas9, and pRdGa5Cas9
UbiKp	AGATGTGGTACCGTGCAGCGTGACCCGGTCGT	construction of intermediate vector pRdUbiCas9
UbiXb	ACTAGGTCTCTCTAGACTGCAGAAGTAACACC AAACAACAGG	construction of intermediate vector pRdUbiCas9
YAOF18	GGCGCGCCTGCAGGTACCTCTGAATCGAGCTT TCGGAA	construction of intermediate vector pRdYCas9
PYao2	ttggtctctctagTCTCTCTCACTCCCTCTTAG	construction of intermediate vector pRdYCas9
Ktrj71	GTCAAGGGTACAACGAGGAATTC	construction of CRISPR/Cas9-mediated *GmNARK* (*Rj7*) gene knockout
Ktrj72	AAACGAATTCCTCGTTGTACCCT	construction of CRISPR/Cas9-mediated *GmNARK* (*Rj7*) gene knockout
KtLjNL1	ATTGGTTGTTCTGATGGATCCTT	construction of CRISPR/Cas9-mediated knockout for targeting *LjNLP4* with pPG35Cas9
KtLjNL2	AAACAAGGATCCATCAGAACAAC	construction of CRISPR/Cas9-mediated knockout for targeting *LjNLP4* with pPG35Cas9, pRd35Cas9 and pRdGa1Cas9
KtLjNL3	GTCATGTTGTTCTGATGGATCCTT	construction of CRISPR/Cas9-mediated knockout for targeting *LjNLP4* with pRd35Cas9 and pRdGa1Cas9
LjNLP4F	GTGGGATCCATATTGGCAAC	PCR amplify *NRSYM1*/*LjNLP4* targeted site for restriction enzyme digestion analysis
LjNLP4R	TACCGTGCTGCCTACAGATG	PCR amplify *NRSYM1*/*LjNLP4* targeted site for restriction enzyme digestion analysis
ktGmR11	GTGTGGTCTCGGTCATAGAATTCATAAAGCTTG AGTTTTAGAGCTAGAAATAGCAAG	construction of p2×35Spro-Cas9-*Rfg1GmNNL1*, pAtGCSpro$_{1200}$-Cas9-*Rfg1GmNNL1* for targeting *Rfg1*

Table S1 All primer sequences used in this paper (to continue).

primers name	Sequence (5'-3')	purposes
ktGmR12	GTGTGGTCTCGAAACCCATGGCATGTTCCTGTTTCAATCTCTTAGTCGACTCTACC	construction of p2×35Spro-Cas9-*Rfg1GmNNL1*, pAtGCSpro1200-Cas9-*Rfg1GmNNL1* for targeting *GmNNL1*
ktLjSNF	GTGTGGTCTCGGTCAGTTGTTCTGATGGATCCTTGTTTTAGAGCTAGAAATAG	construction of p*2×35Spro*-Cas9-*LjNLP4LjSYMRK*, p*AtGCSpro*1200-Cas9-*LjNLP4LjSYMRK* for targeting *LjNLP4*
ktLjSNR	GACAGGTCTCGAAACTGCAGTTCCTCTTACTTCACAATCTCTTAGTCGACTCT	construction of p*2×35Spro*-Cas9-*LjNLP4LjSYMRK*, p*AtGCSpro*1200-Cas9-*LjNLP4LjSYMRK* for targeting *LjSYMRK*
ktSlTRY1	CTGTGGTCTCGGTCAGGTGGTGCATGAGTTTGTGTGTTTTAGAGCTAGAAATAGC	construction of p2×35Spro-Cas9-*SlTRY* and pAtGCSpro1200-Cas9-*SlTRY*
ktSlTRY2	CTGTGGTCTCGAAACACAAGTTTGTGCATCCTGTCAATCTCTTAGTCGACTCTAC	construction of p2×35Spro-Cas9-*SlTRY* and pAtGCSpro1200-Cas9-*SlTRY*
Rj71	ACTTGAGGGCACTGCAGACT	PCR amplify *GmNARK* (*Rj7*) targeted site for restriction enzyme digestion analysis
Rj72	ACGTCTTCGCAAGGTTCATC	PCR amplify *GmNARK* (*Rj7*) targeted site for restriction enzyme digestion analysis
GmRHin1	TCTAATTCGAGACACAGGCAG	PCR amplify *GmNNL1* targeted site for restriction enzyme digestion analysis
GmRHin2	CTATTTGGCAGATGCTCGG	PCR amplify *GmNNL1* targeted site for restriction enzyme digestion analysis
GmRNco1	TTGTTGCACCCATTCGATC	PCR amplify *Rfg1* targeted site for restriction enzyme digestion analysis
GmRNc4	GCCCCTTATATACTTCGAATCCA	PCR amplify *Rfg1* targeted site for restriction enzyme digestion analysis
LjSYF	GTGAACTTGACTACAGGGGAAC	PCR amplify *LjSYMRK* targeted site for restriction enzyme digestion analysis
LjSYR	CCTGCATAGGGATAAATGTCAG	PCR amplify *LjSYMRK* targeted site for restriction enzyme digestion analysis
SLTRY1	CCCTCCTAATCAACAGCAACTCTC	PCR amplify *SlTRY* targeted site for restriction enzyme digestion analysis
SLTRY2	GGCCAACAAGTTCATTCGATG	PCR amplify *SlTRY* targeted site for restriction enzyme digestion analysis
GmActinF	GAGCTATGAATTGCCTGATGG	qRT-PCR primer for amplification *Actin* gene in soybean
GmActinR	CGTTTCATGAATTCCAGTAGC	qRT-PCR primer for amplification *Actin* gene in soybean
GUSPF	ATGGTAGATCTGAGGAACCG	qRT-PCR primer for amplification *GUSPlus* gene
GUSPR	GCCAATGTCATTGTAACTGC	qRT-PCR primer for amplification *GUSPlus* gene
