# Supplementary material for: AtGCS promoter-driven clustered regularly interspaced short palindromic repeats/Cas9 highly efficiently generates homozygous/biallelic mutations in the transformed roots by Agrobacterium rhizogenes–mediated transformation
Source: Front Plant Sci. 2022 Oct 18;13:952428. doi: 10.3389/fpls.2022.952428 (PMC9623429; doi:10.3389/fpls.2022.952428)
Supplement: Table S2 — Comparison of pAtGCSpro1178-Cas9-Rj7, pUbiqutinpro-Cas9-Rj7, pYAOpro-Cas9-Rj7, and p2×35Spro-Cas9-Rj7 genome editing efficiency in soybean hairy roots. [file Table_2.pdf]

Table S2. Comparison of p*AtGCSpro*$_{1178}$-Cas9-*Rj7*, p*Ubiqutin*$_{pro}$-Cas9-*Rj7*, p*YAO*$_{pro}$-Cas9-*Rj7*, and p2×*35Spro*-Cas9-*Rj7* genome editing efficiency in soybean hairy roots

Cas9 system	no. of H/BM roots/ no. of roots examined	H/BM rate (%)
p*2×35Spro*-Cas9-*Rj7*	17/30	56.7 %
p*Ubiqutin*$_{pro}$-Cas9-*Rj7*	4/30	13.3 %
p*YAO*$_{pro}$-Cas9-*Rj7*	4/30	13.3 %
p*AtGCSpro*$_{1178}$-Cas9-*Rj7*	21/30	70.0 %
