# Supplementary material for: AtGCS promoter-driven clustered regularly interspaced short palindromic repeats/Cas9 highly efficiently generates homozygous/biallelic mutations in the transformed roots by Agrobacterium rhizogenes–mediated transformation
Source: Front Plant Sci. 2022 Oct 18;13:952428. doi: 10.3389/fpls.2022.952428 (PMC9623429; doi:10.3389/fpls.2022.952428)
Supplement: Table S3 — Comparison of pAtGCSpro1178-Cas9-LjNLP4, pUbiqutinproCas9-LjNLP4, pYAOpro-Cas9-LjNLP4, and p2×35Spro-Cas9-LjNLP4 genome editing efficiency in L. japonicus hairy roots. [file Table_3.pdf]

Table S3. Comparison of p*AtGCSpro*$_{1178}$-Cas9-*LjNLP4*, p*Ubiqutin*$_{pro}$-Cas9-*LjNLP4*, p*YAO*$_{pro}$-Cas9-*LjNLP4*, and p2×*35Spro*-Cas9-*LjNLP4* genome editing efficiency in *L. japonicus* hairy roots

Cas9 system	no. of H/BM roots/ no. of roots examined	H/BM rate (%)
p*2×35Spro*-Cas9-*LjNLP4*	5/16	31.3 %
p*Ubiqutin*$_{pro}$-Cas9-*LjNLP4*	3/24	12.5 %
p*YAO*$_{pro}$-Cas9-*LjNLP4*	4/24	16.7 %
p*AtGCSpro*$_{1178}$-Cas9-*LjNLP4*	13/16	81.3 %
